# RBM25 Regulates p38 MAPK Pathway Activation via Exon 16 Skipping of MAP4K4 in a Rat Model of Post‐Infarction Heart Failure

**DOI:** 10.1096/fba.2025-00201

**Published:** 2025-12-16

**Authors:** Hao Li, Keyi Zhang, Chen Liu, Xin Tian, Guangli Zhou, Wanshu Liu, Yang Li, Lingmin Zhao, Luqiao Wang, Ping Yang

**Affiliations:** ^1^ Cardiovascular Clinical Medicine Center The First Affiliated Hospital of Kunming Medical University Kunming China; ^2^ Department of Radiology Affiliated Hospital of Yunnan University Kunming China; ^3^ School of Public Health Kunming Medical University Kunming China

**Keywords:** alternative splicing, cardiomyocyte apoptosis, heart failure, MAP4K4, MAPK signaling pathway, RBM25

## Abstract

Ischemic cardiomyopathy remains a leading cause of heart failure (HF), yet its molecular mechanisms remain incompletely defined. This study aimed to identify the RNA‐binding protein 25(RBM25) as a critical regulator of HF progression through MAP4K4 alternative splicing and p38 MAPK pathway activation. A left anterior descending (LAD) coronary artery ligation‐induced HF model was established in Sprague–Dawley (SD) rats, with pericardial delivery of lentiviral vectors for RBM25 overexpression (OE‐RBM25) or shRNA‐mediated knockdown (sh‐RBM25). Quantitative PCR (qPCR) experiments confirmed that overexpression of RBM25 induces exon 16 skipping in MAP4K4. Computational modeling further predicted that the resulting variant enhances binding to MAP3K1 and potentially activates the MAPK pathway. Cardiac function, infarct size, apoptosis, and molecular markers were evaluated via echocardiography, TTC staining, ELISA, qPCR, Western blot, and TUNEL assays. RBM25 overexpression significantly increased myocardial infarction area compared to the HF control group (*p* < 0.01), while RBM25 knockdown reduced infarct size (*p* < 0.01). Consistently, RBM25 overexpression upregulated pro‐apoptotic markers (Caspase‐3, Bax; *p* < 0.05) and downregulated anti‐apoptotic Bcl‐2 (*p* < 0.05), whereas RBM25 inhibition reversed these effects. Mechanistically, RBM25 induced exon 16 skipping in MAP4K4, generating a truncated isoform that activated MAPK signaling, as evidenced by increased phosphorylation of ERK (*p* < 0.05) and elevated downstream effectors (C‐FOS, EGR1, PARP1; *p* < 0.05). P38 MAPK inhibition (SB203580) attenuated RBM25‐mediated myocardial injury, while agonist‐induced MAPK activation (Gambogic Amide) abolished the protective effects of RBM25 knockdown. These findings suggest that RBM25 exacerbates HF through MAP4K4 splicing‐dependent p38 MAPK activation, highlighting its potential as a therapeutic target for ischemic cardiomyopathy.

## Graphical abstract

This graphical abstract illustrates how RBM25 exacerbates post‐infarction heart failure in rats by promoting exon 16 skipping in MAP4K4, generating a truncated isoform (MAP4K4‐Δ16) that enhances MAP3K1 binding and activates the p38 MAPK pathway, leading to increased cardiomyocyte apoptosis via upregulated Caspase‐3 and Bax and downregulated Bcl‐2.

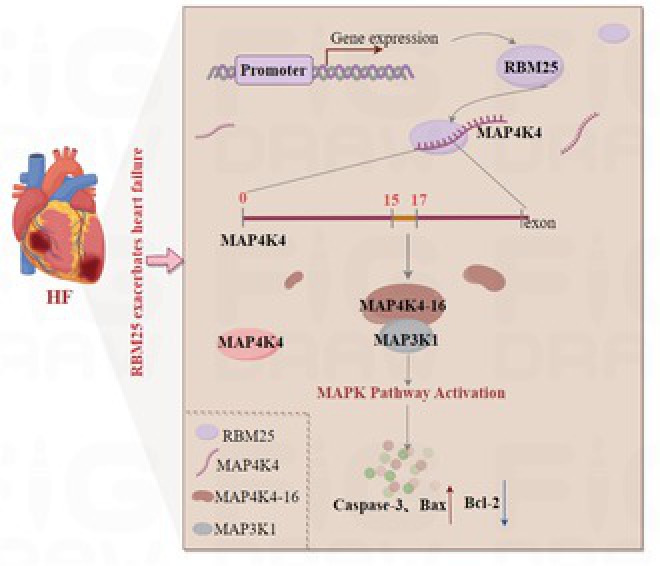

## 1 Introduction

Ischemic cardiomyopathy persists as the principal etiology of HF, a global health crisis affecting approximately 64.3 million individuals with staggering mortality rates–67% within 5 years of diagnosis. Demographic shifts, including population aging and escalating obesity rates, continue to drive HF prevalence upward [1, 2, 3]. The pathophysiological cascade involves ischemia/reperfusion injury, adverse ventricular remodeling, and progressive cardiomyocyte apoptosis/necrosis [4]. Despite therapeutic advancements in percutaneous interventions, pharmacological regimens, and mechanical circulatory support devices, ischemic cardiomyopathy remains the leading indication for cardiac transplantation [5]. This underscores an urgent need to elucidate molecular mechanisms underlying HF progression for targeted therapeutic development.

RBPs serve as primary regulators of post‐transcriptional gene expression through splicing modulation, RNA stabilization, and translational control. Their dysregulation is increasingly implicated in cardiovascular pathologies, where aberrant RBP activity exacerbates cardiomyocyte apoptosis and fibrotic remodeling. Among RBPs, RBM25 harbors three functionally conserved domains: (1) an RNA recognition motif (RRM) enabling sequence‐specific RNA binding, (2) a serine/arginine‐rich (SR) domain mediating spliceosome interactions, and (3) a C‐terminal domain facilitating nuclear localization and protein partnerships [6]. Through these structural elements, RBM25 governs alternative splicing (AS) events—particularly exon skipping (ES)—that critically influence protein diversity [7]. Emerging evidence links RBM25 dysregulation to cardiac pathologies via endoplasmic reticulum stress potentiation through CHOP signaling and aberrant ion channel splicing [8, 9, 10, 11]. Notably, recent studies position RBPs as key modulators of stress‐responsive pathways, including the mitogen‐activated protein kinase (MAPK) cascade [12], suggesting RBM25 may orchestrate MAPK‐mediated myocardial injury.

The MAPK signaling pathway integrates cellular responses to oxidative stress, inflammation, and apoptosis through three evolutionarily conserved subfamilies: extracellular signal‐regulated kinases (ERK), c‐Jun N‐terminal kinases (JNK), and p38 kinases [13]. Moreover, apoptosis is essential for the development of HF and post‐infarction cardiac remodeling [14]. Clinical analyses reveal persistent MAPK activation in infarct border zones, correlating with apoptotic cardiomyocyte depletion and maladaptive remodeling [15]. Central to this cascade is mitogen‐activated protein kinase 4 (MAP4K4), which phosphorylates MAP3Ks to initiate downstream MAPK signaling. Elevated MAP4K4 expression in failing human myocardium [16] and its regulation by apoptosis‐modulating miRNAs (e.g., miR‐30d) highlight its pathological role [17], though upstream regulatory mechanisms remain elusive. Post‐transcriptional control through non‐coding RNAs, RBPs, and alternative splicing likely regulate MAP4K4 activity in cardiac contexts.

Our preliminary RNA sequencing of RBM25‐overexpressing H9c2 cardiomyocytes revealed a prominent ES event at exon 16 of MAP4K4 transcripts. Given RBM25's splicing regulatory capacity and MAP4K4's established role in MAPK activation, we hypothesize that RBM25 drives HF progression through ES‐mediated generation of a truncated MAP4K4 isoform, thereby hyperactivating pro‐apoptotic MAPK signaling. This mechanism positions RBM25 as a novel therapeutic target for mitigating ischemic cardiomyopathy.

## 2 Methods and Materials

### 2.1 Establishment of Heart Failure Models in **SD** Rats

Healthy SD rats (120–140 g; males 8–15 weeks, females 6–12 weeks; Sibefu Biotechnology, Beijing) were used to establish a heart failure (HF) model via LAD coronary artery ligation, as described previously [18]. Rats were anesthetized with isoflurane, and LAD was ligated 1–2 mm below the left atrial appendage. Successful ligation was confirmed by myocardial blanching and ECG ST‐segment elevation. Sham‐operated rats underwent thoracotomy without ligation. All procedures followed the 3R principles and were approved by the Animal Experiment Ethics Review Committee of Kunming Medical University (kmmu20211238).

### 2.2 Experimental Grouping and Interventions

Surviving rats (*n* = 48) were randomly allocated into eight groups (*n* = 6 each): sham, HF, OE‐NC (empty vector overexpression), OE‐RBM25 (RBM25 overexpression), OE‐RBM25 + SB203580 (p38 MAPK inhibitor), sh‐NC (scramble shRNA), sh‐RBM25 (RBM25 knockdown), sh‐RBM25 + Gambogic Amide (MAPK inducer). Lentiviral vectors (2 × 10^9^ TU/mL, 200 μL) were injected into the pericardial cavity under echocardiographic guidance, administered once every 4 weeks for a total of two injections. SB203580 (5 mg/kg/day; MEC, RWJ 64809) or Gambogic Amide (2 mg/kg/day; Alomone Labs, G‐235) was injected intraperitoneally daily for 8 weeks. The sham and HF groups received equivalent saline. Rats were maintained on standard chow with ad libitum water access. After the 8‐week intervention, rats were euthanized via exsanguination under deep anesthesia, and heart tissues and serum samples were collected for subsequent analysis, with researchers blinded to group assignments during data collection.

### 2.3 Echocardiographic Evaluation

Transthoracic echocardiographic measurements were performed on all rats using a Philips EPIQ 7C ultrasound machine (Royal Philips of the Netherlands Inc., Amsterdam, Netherlands) equipped with an L12‐3 phased array linear transducer. Continuous cardiac structure and function assessment was conducted at baseline (pre‐surgery) and at 4‐ and 8‐week post‐surgery. Parameters measured included left ventricular end‐systolic diameter (LVESD), left ventricular end‐diastolic diameter (LVEDD), left ventricular ejection fraction (LVEF), and left ventricular fractional shortening (LVFS). All measurements were performed under light anesthesia (1.5% isoflurane) with heart rate maintained at 450–550 bpm via ECG monitoring. These measurements provided a quantitative assessment of left ventricular systolic function and contractility.

### 2.4 Cardiac TTC Staining

Hearts were excised, immersed in phosphate‐buffered saline (PBS) at 0°C–4°C, frozen at −20°C for 30 min, and sliced into 2 mm transverse sections using a slicing matrix. Sections were incubated in 2% 2,3,5‐triphenyl tetrazolium chloride (TTC) solution at 37°C pH 7.4 for 30 min. After rinsing with PBS, sections were photographed and fixed in 10% neutral formalin. Infarct area was analyzed using a pathological image analysis system by two blinded operators, calculating infarct volume as infarct area × slice thickness and infarct percentage as infarcted area/total left ventricular area.

### 2.5 ELISA Quantitative Analysis of NT‐Pro BNP, CRP, IL‐6, and TNF‐α

Serum levels of NT‐pro BNP, CRP, IL‐6, and TNF‐ɑ were measured using ELISA kits (MEIMIAN), with cross‐reactivity < 1% and LLOD values of 5 pg/mL (NT‐pro BNP), 0.2 ng/mL (CRP), 1.5 pg/mL (IL‐6), and 2 pg/mL (TNF‐ɑ). Samples were centrifuged at 2000 × *g* for 15 min at 4°C within 30 min of collection and stored at −80°C in aliquots. Standards and samples were assayed in triplicate (CV < 8%), with OD measured at 450 nm using a microplate reader.

### 2.6 Quantitative PCR Analysis

Total RNA was extracted from myocardial tissues of each experimental group and reverse‐transcribed into complementary DNA (cDNA) using a standardized reverse transcription protocol. qPCR was performed using the 2 × Universal Blue SYBR Green qPCR Master Mix (Servicebio, BZ2106008). The thermal cycling conditions were as follows: an initial denaturation at 95°C for 1 min, followed by 40 cycles of denaturation at 95°C for 20 s, annealing at 55°C for 20 s, and extension at 72°C for 30 s. The primer sequences used for qPCR amplification are presented in Table S1.

### 2.7 Western Blotting Analysis

Myocardial tissues were homogenized in lysis buffer, centrifuged, and protein concentration determined using BCA assay (Beyotime). 80 μg protein per sample was separated by SDS‐PAGE, transferred to PVDF membranes, blocked, and incubated overnight at 4°C with primary antibodies: RBM25 (1:2000), Caspase‐3 (1:2000), Bax (1:1000), Bcl‐2 (1:1000), CSF1 (1:5000), ERK (1:1000), p‐ERK (1:1000), c‐FOS (1:1000), EGR1 (1:1000), PARP1 (1:1000), GAPDH (1:5000). Membranes were then incubated with HRP‐conjugated secondary antibodies. Signals were visualized using a Bio‐Rad ChemiDoc XRS+ system and quantified with ImageJ software by normalizing target band intensity to the internal control.

### 2.8 TUNEL Assay for Detection of Cardiomyocyte Apoptosis

Paraffin‐embedded myocardial sections were deparaffinized, rehydrated, treated with Proteinase K (20 μg/mL), and incubated with TUNEL reaction mixture (Servicebio G1502‐50T) at 37°C for 1 h. Sections were washed with PBS, stained with DAPI, and imaged under fluorescence microscopy (3–5 fields per section at 400× magnification). Apoptotic index (%) = number of apoptotic cells (red fluorescence)/total cells (blue fluorescence) × 100%.

### 2.9 Immunohistochemical Analysis

Paraffin‐embedded myocardial sections were deparaffinized, hydrated, antigen‐retrieved with citrate buffer, blocked with 3% H_2_O_2_, and incubated overnight at 4°C with primary antibodies: RBM25 (1:50), Caspase‐3 (1:300), Bax (1:300), Bcl‐2 (1:50), MAP4K4 (1:50), ERK (1:100), P‐ERK (1:50), C‐FOS (1:50), PARP1 (1:50), EGR1 (1:100). Sections were then incubated with HRP‐conjugated secondary antibodies, developed with DAB, counterstained with hematoxylin, dehydrated, and mounted. Quantitative analysis was performed using ImageJ, calculating average optical density (AOD) as cumulative optical density/field area.

### 2.10 AlphaFold3 Structural Prediction

Protein sequences of wild‐type MAP4K4 (UniProt ID: O95819), exon 16‐deleted MAP4K4 isoform (ΔEx16), and MAP3K1 (UniProt ID: Q13233) were retrieved from UniProt. Tertiary structures were predicted using AlphaFold3 [19] with default parameters. Five models were generated per protein, and the highest‐ranked model by predicted local distance difference test (pLDDT) score was selected for downstream analysis.

### 2.11 HDOCK Molecular Docking Simulations

Structural preprocessing was performed sequentially: Protonation State Assignment: Structures were processed through H++ 3.0 web server [20] at Parameterization: physiological protonation states. Charge Parameterization: Using UCSF Chimer [21], protein Amber ff14SB force field charges were assigned. Protein–protein docking was executed on the HDOCK server [22]. Using FFT‐based rigid‐body mode and ITScorePP scoring. Top poses were analyzed for binding interfaces using PyMOL 2.5.7 and LigPlot+ v.2.2.5 [23].

### 2.12 Statistical Analysis

Data analysis was conducted using GraphPad Prism software. All experimental results were presented as mean ± standard error of the mean (Mean ± SEM). The t‐test was applied for pairwise comparisons, while one‐way analysis of variance (ANOVA) was used for evaluating differences among multiple groups. Post hoc tests were performed using Tukey's HSD method to determine specific differences between groups. Statistical significance was defined as *p* < 0.05. Additionally, Pearson correlation analysis was used to assess relationships among variables from individual sample data (*n* = 6 per group, excluding sham and drug‐intervention groups: HF, OE‐NC, OE‐RBM25, sh‐NC, sh‐RBM25). Variables included: (1) RBM25 protein levels (from WB, normalized to GAPDH); (2) ΔEx16 ratio (quantified from qPCR gel band intensities using ImageJ software); (3) p‐ERK levels (from WB, normalized to GAPDH). Statistical significance was defined as *p* < 0.05 for all analyses.

Sample size (*n* = 6 rats per group) was determined based on preliminary experiments considering ethical constraints and resource availability. To address potential power concerns, a post hoc analysis in G*Power (v3.1.9.7) was performed under the assumptions of normal distribution and homogeneity of variance. The results showed low power for small‐to‐medium effects (power < 0.50 for Cohen's *d* ≤ 0.5 in t‐tests; power < 0.40 for Cohens *f* ≤ 0.25 in ANOVA), but moderate‐to‐sufficient power for large effects (≈0.62 for Cohen's *d* = 0.8 in two‐tailed *t*‐tests; ≈0.75 for Cohen's *f* = 0.4 in ANOVA). This indicates adequate power to detect robust differences but potential underpowering for subtler effects, which is noted as a limitation of the current study.

Detailed protocols for the methods outlined above are available in the Supporting Information, including the HF model establishment in SD rats (Methods S1), grouping and interventions (Methods S2), TTC staining (Methods S3), ELISA quantitative analysis (Methods S4), Western blotting (Methods S5), TUNEL assay for cardiomyocyte apoptosis (Methods S6), and HDOCK molecular docking simulations (Methods S7). These provide expanded procedural steps, reagent details, and parameters.

## 3 Results

### 3.1 Preliminary Finding on RBM25's Regulation of Alternative Splicing in the MAPK Pathway

In our previous study [9], overexpression of RBM25 in rat cardiomyocytes (H9C2) was investigated using RNA‐seq and improved RNA immunoprecipitation sequencing (iRIP‐seq) to identify bound and regulated target genes. The raw sequencing data from this study have been deposited in the NCBI Gene Expression Omnibus (GEO) under accession numbers GSE209940 (RNA‐seq) and GSE209941 (iRIP‐seq). KEGG pathway enrichment analysis of differentially expressed genes (DEGs) and AS events revealed significant enrichment in pathways related to immune and inflammatory responses, including the MAPK signaling pathway. Overlap analysis between RNA‐seq and iRIP‐seq data identified key targets such as Csf1 (also known as CSF1), which is bound by RBM25 and undergoes regulated AS, contributing to inflammatory processes. Re‐analysis of this RNA‐seq data further confirmed that RBM25 significantly regulates ES events in the MAP4K4 gene. These bioinformatics insights provide foundational evidence for RBM25's role in modulating AS events within the MAPK pathway, potentially influencing cardiomyocyte apoptosis in the context of HF (Figure S1).

### 3.2 RBM25‐Mediated Exon 16 Skipping of MAP4K4 Activates the MAPK Signaling Pathway

**FIGURE 1:**
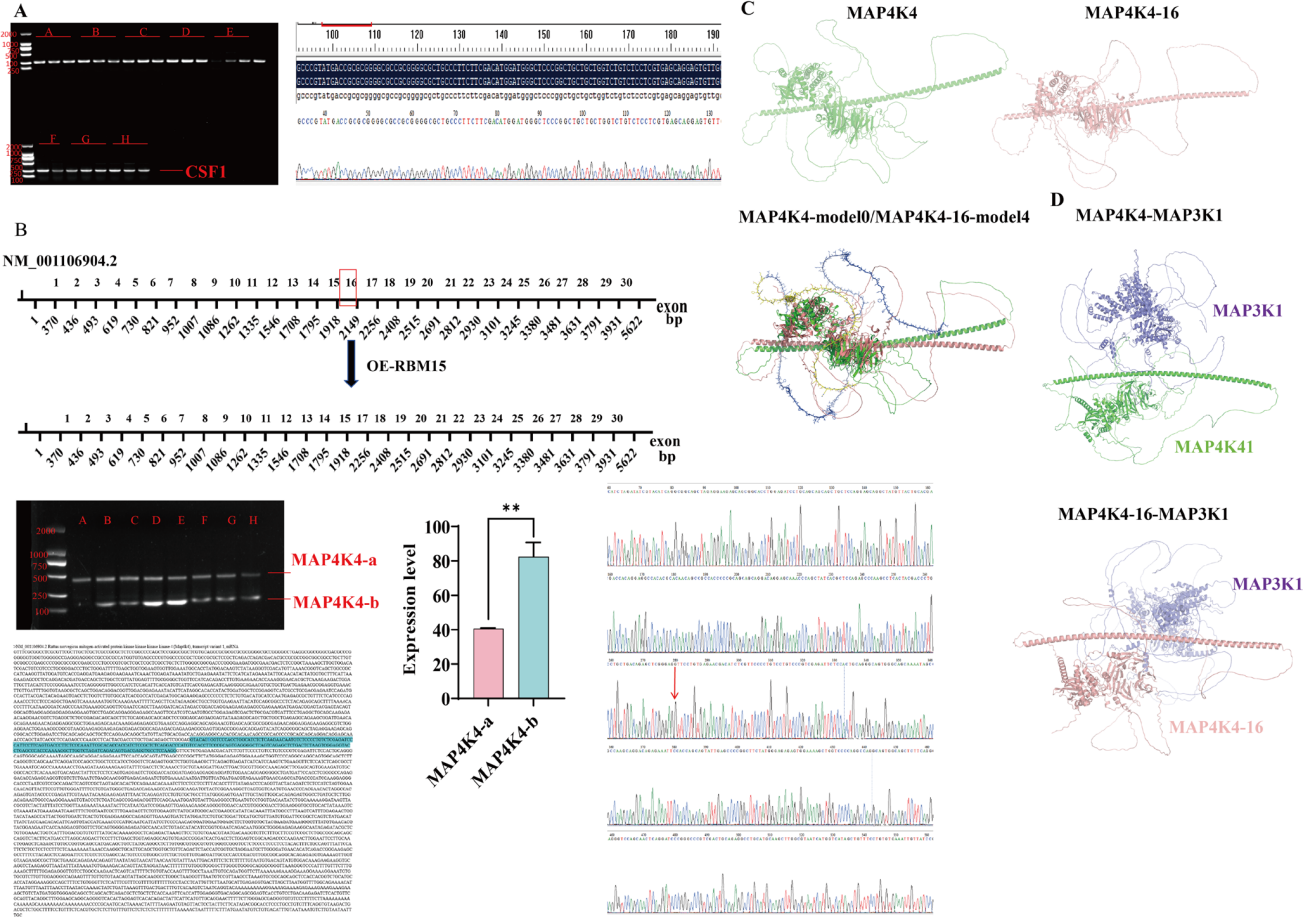
RBM25 regulates exon 16 skipping of MAP4K4. (A) PCR amplification of CSF1; (B) PCR amplification of MAP4K4‐a/b. The gel image shows the following groups: A: Sham, B: HF(heart failure model), C: OE‐NC(empty vector overexpression), D: OE‐RBM25(RBM25 overexpression), E: E‐RBM25 + SB203580(p38 inhibitor), F: sh‐NC (scramble shRNA), G: sh‐RBM25 (RBM25 knockdown), H: sh‐RBM25 + Gambogic Amide MAPK activation; (C) Structural Consequences of ΔEx16 Cartoon representations of AlphaFold3‐predicted structures: Wild‐type MAP4K4(Green); Exon 16‐deficient MAP4K4‐ΔEx16 (Pink).ΔEx16 induces conformational rearrangement in the kinase domain (circled region); (D) Protein–protein docking simulations via HDOCK. Predicted binding interfaces and quantified affinities between MAP3K1 (Purple) and wild‐type MAP4K4 (Green) versus MAP4K4‐ΔEx16 (Pink).

To investigate the role of RBM25 in MI and HF via modulation of the MAPK signaling pathway, we established an SD rat HF model. Cardiac‐specific overexpression and inhibition of RBM25 were achieved using lentiviral vectors. Successful viral transduction and functional efficacy of both RBM25 knockdown and overexpression vectors were confirmed (Figure S2). We next examined whether RBM25 regulates alternative splicing of MAP4K4, specifically exon 16 skipping, thereby generating transcripts that encode proteins capable of activating MAPK signaling and exacerbating HF. To determine whether RBM25 mediates MAP4K4 exon 16 skipping, qPCR was performed on myocardial tissues from eight experimental rat groups using specific primers amplifying regions within the coding sequences (CDS) of CSF1 and MAP4K4 genes. The resulting PCR products were sequenced to confirm transcript integrity and identify splicing variants. The results showed that CSF1 consistently produced a single amplification band across all eight experimental groups, and sequencing confirmed that the amplified sequence was identical to the reference sequence in the NCBI database (Figure 1A). In contrast, MAP4K4 exhibited two distinct amplification products in all experimental groups except the sham group, indicating the presence of two alternative splicing variants: MAP4K4‐a and MAP4K4‐b. Sequencing analysis further revealed that MAP4K4‐a was identical to the NCBI reference sequence (NM_001106904.2), whereas MAP4K4‐b was characterized by the skipping of exon 16 compared to the reference sequence (Figure 1B). These results show that RBM25 exclusively promotes alternative splicing of MAP4K4, which leads to exon 16 skipping, but has no effect on alternative splicing of CSF1.

Structural predictions generated by AlphaFold3 revealed distinct tertiary architectures between wild‐type MAP4K4 and the exon 16‐deficient isoform (MAP4K4‐ΔEx16) (Figure 1C). Comparative analysis demonstrated significant conformational divergence in the C‐terminal regulatory domain. Protein–protein docking simulations via HDOCK (Figure 1D) quantified binding affinities between MAP3K1 and both isoforms. The MAP4K4‐ΔEx16: MAP3K1 complex exhibited enhanced binding affinity (docking score: −142.5 ± 3.2) compared to wild‐type MAP4K4: MAP3K1 (−100.3 ± 4.1, *p* < 0.001, Table S2), indicating stabilization through exon skipping‐induced structural reorganization. Our data demonstrate that RBM25‐mediated alternative splicing specifically targets MAP4K4 through exon 16 skipping (ΔEx16). Critically, the resultant MAP4K4‐ΔEx16 isoform acquires enhanced binding capacity to MAP3K1, suggesting a gain‐of‐function mechanism in signal transduction pathways.

Pearson correlation analysis revealed positive associations: RBM25 protein levels vs. ΔEx16 ratio (*r* = 0.5890, *p* = 0.0008), ΔEx16 ratio vs. p‐ERK levels (*r* = 0.5928, *p* = 0.0008), and RBM25 protein levels vs. p‐ERK levels (*r* = 0.8391, *p* < 0.0001; Figure S3).

### 3.3 RBM25 Regulates the Expression of MAP4K4, ERK, C‐FOS, EGR1, PARP1 to Promote Heart Failure

**FIGURE 2:**
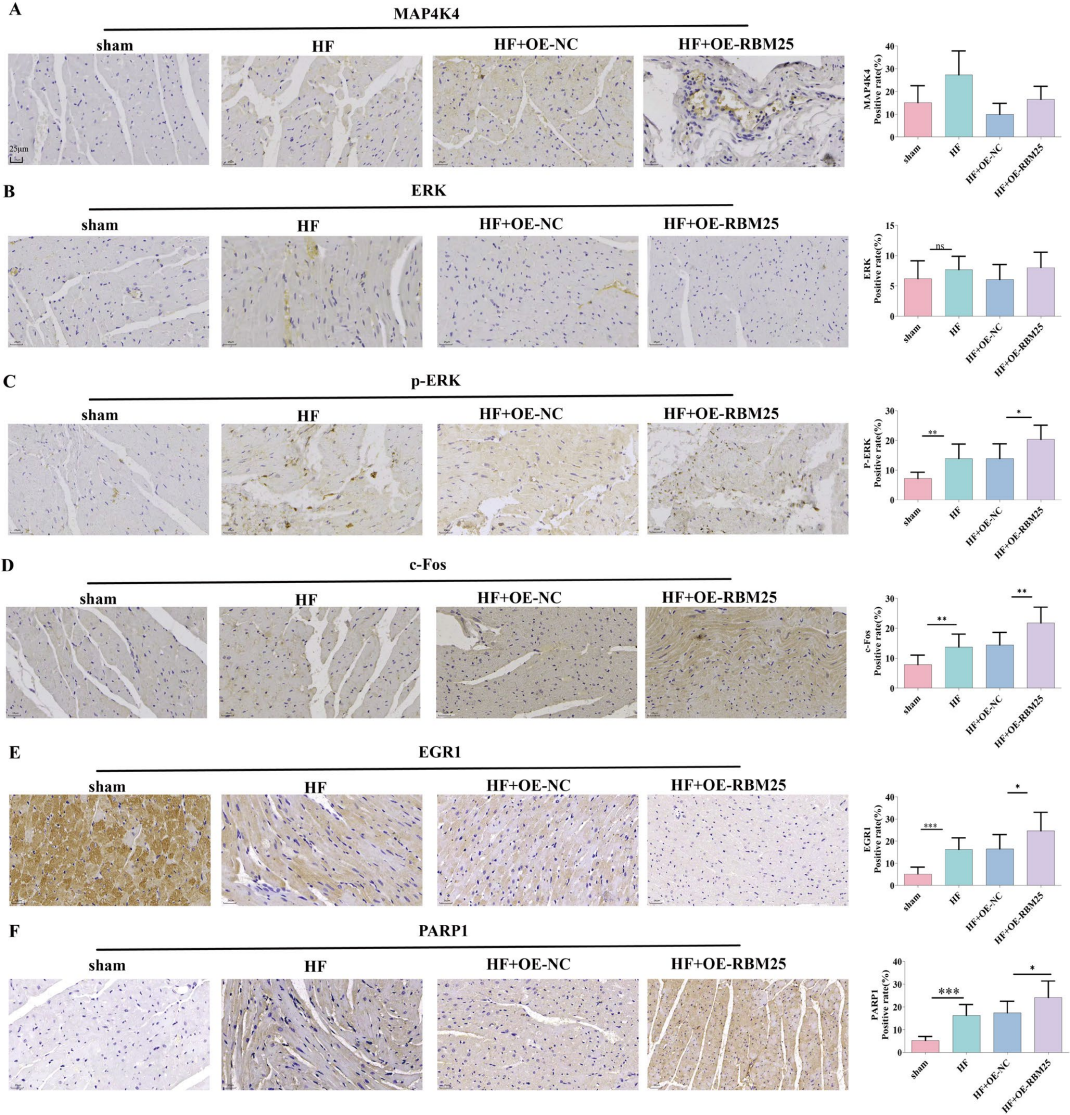
IHC was performed to assess expression levels of: (A) MAP4K4, (B) ERK, (C) p‐ERK, (D) c‐FOS, (E) EGR1, and (F) PARP1 in cardiac tissue sections. All images were captured at 400× magnification with scale bars = 25 μm. Statistical significance thresholds: Ns (*p* > 0.05), *p < 0.05, **p < 0.01, ***p < 0.001.

To determine whether RBM25‐mediated exon skipping in MAP4K4 activates the MAPK pathway, IHC analysis was performed to assess expression of MAP4K4, p‐ERK, total ERK, c‐FOS, EGR1, and PARP1 in cardiac tissues from the following experimental groups: 1. Sham‐operated controls; 2. HF model group; 3.0HF model + empty vector overexpression; 4. HF model + RBM25 overexpression (Figure 2). Compared with the control group, the HF model group exhibited significantly increased expression levels of MAP4K4, p‐ERK, c‐FOS, EGR1, and PARP1 (*p* < 0.05). Subsequent RBM25 overexpression intervention in HF models further elevated the expression of these factors in cardiac tissues. These results indicate that RBM25‐mediated alternative splicing (exon 16 skipping of MAP4K4) enhances MAPK pathway activation.

### 3.4 RBM25 Positively Regulates p38 MAPK Signaling to Exacerbate Myocardial Infarction

**FIGURE 3:**
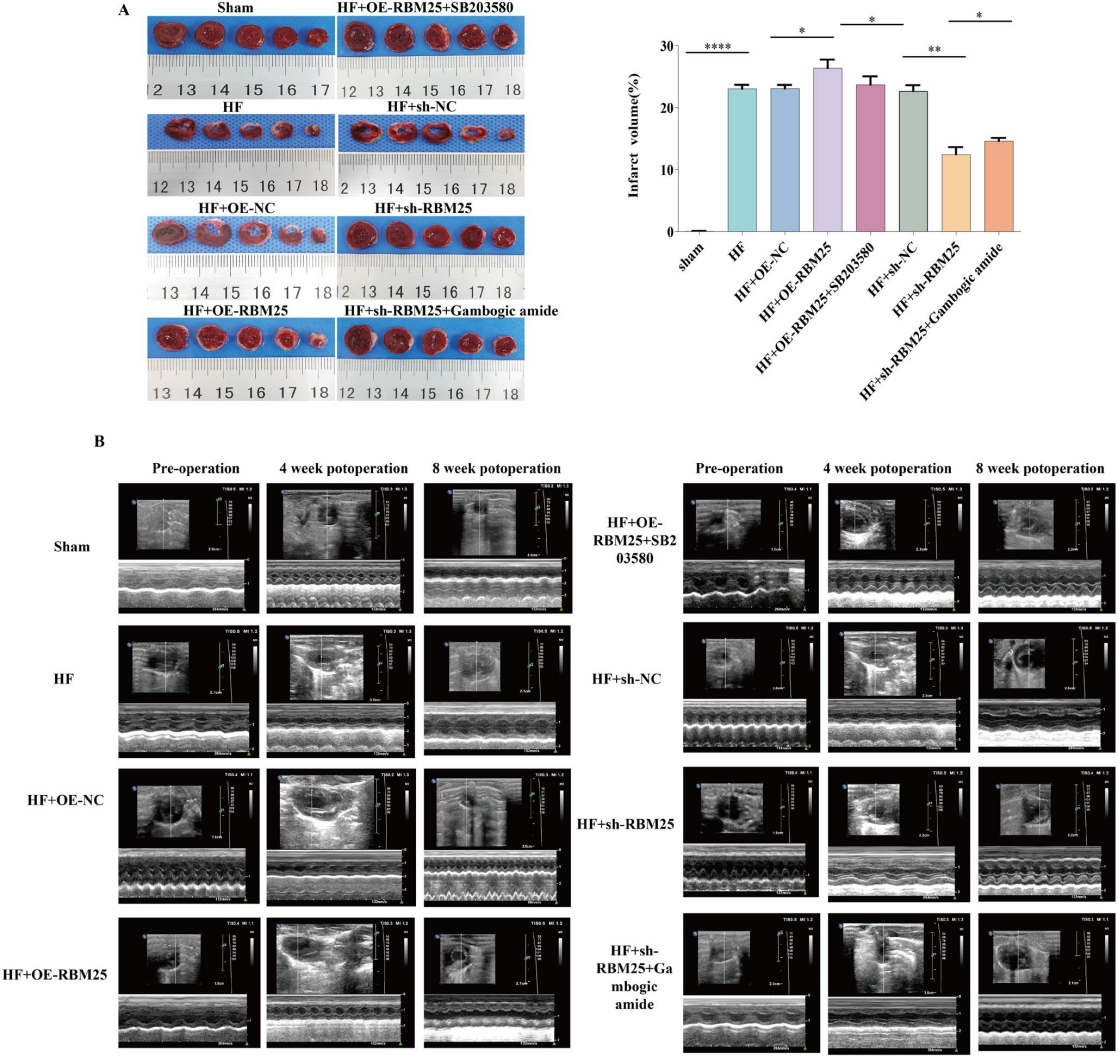
RBM25 positively regulates MAPK signaling to exacerbate myocardial infarction. (A) Gross specimen images from TTC staining and statistical chart of myocardial infarction area; (B) echocardiographic measurements were performed to assess the left ventricular end‐systolic diameter (LVESD), left ventricular end‐diastolic diameter (LVEDD), and left ventricular ejection fraction (LVEF) in rats from each group. Statistical significance is indicated as follows: Ns (not significant) *p* > 0.05; *p < 0.05; **p < 0.01; ***p < 0.001; ****p < 0.0001.

Previous experimental results have demonstrated that RBM25 activates the MAPK signaling pathway by regulating exon skipping of MAP4K4. To further investigate whether RBM25 exacerbates myocardial infarction and thereby promotes the progression of HF through this mechanism, we performed TTC staining on cardiac tissues from eight groups of rats (Figure 3A). The results revealed a significantly larger myocardial infarction area in the HF model group compared to the sham group (*p* < 0.0001, Figure 3A), confirming the successful establishment of the LAD coronary artery ligation model.

Compared to the HF‐OE‐NC group, overexpression of RBM25 (HF + OE‐RBM25 group) resulted in a marked increase in myocardial infarction area (*p* < 0.01). Conversely, inhibition of RBM25 (HF + sh‐RBM25 group) led to a significant reduction in myocardial infarction area compared to the HF + sh‐NC group (*p* < 0.01, Figure 3A). These findings indicate that RBM25 overexpression aggravates myocardial infarction, whereas its downregulation exerts a protective effect.

Further analysis demonstrated that administration of the p38 MAPK inhibitor SB203580 (HF + OE‐RBM25 + SB203580 group) significantly reduced the myocardial infarction area compared to the HF + OE‐RBM25 group (*p* < 0.05). In contrast, indirect activation of the MAPK pathway in the HF + sh‐RBM25 + Gambogic Amide group significantly increased the myocardial infarction area compared to the HF + sh‐RBM25 group (*p* < 0.05, Figure 3A). These results suggest that p38 MAPK inhibition attenuates the pro‐infarction effects of RBM25 overexpression, while MAPK indirect activation counteracts the protective effects of RBM25 knockdown.

Echocardiography was performed preoperatively and at 4‐ and 8‐week post‐surgery to evaluate cardiac function, including LVESD, LVEDD, LVEF, and LVFS (Figure 3B). The measurements are presented in Table S3. In the sham group, within‐group changes in LVEDD, LVESD, LVEF, and LVFS across time points did not reach statistical significance (*p* > 0.05). In contrast, the HF group exhibited ventricular dilation and functional decline: increased LVEDD/LVESD and decreased LVEF/LVFS (*p* < 0.05 vs. baseline and sham), confirming model success.

Most intervention groups (HF, OE‐NC, OE‐RBM25, OE‐RBM25 + SB203580, sh‐NC, sh‐RBM25, sh‐RBM25 + Gambogic Amide) displayed worsened parameters vs. sham (*p* < 0.05), except LVFS at 8 weeks in sh‐RBM25 and LVEF/LVFS at 4 weeks in OE‐RBM25 + SB203580. OE‐NC mirrored HF with no differences (*p* > 0.05) vs. OE‐NC, OE‐RBM25 worsened outcomes: elevated LVEDD/LVESD and reduced LVEF/LVFS (*p* < 0.05 vs. baseline, sham, and OE‐NC, except LVEDD at 4 weeks vs. OE‐NC *p* > 0.05). SB203580 partially reversed: decreased LVESD and increased LVEF/LVFS vs. OE‐RBM25 (*p* < 0.05; *p* < 0.05 vs. baseline for all, vs. sham for LVEDD/LVESD at 4 weeks and all at 8 weeks, except LVEF/LVFS at 4 weeks).

sh‐NC resembled HF (*p* < 0.05 vs. baseline/sham; *p* > 0.05 vs. HF). In sh‐RBM25 vs. sh‐NC: at 4 weeks, LVEDD unchanged (*p* > 0.05 vs. baseline/sh‐NC, *p* < 0.05 vs. sham), LVESD increased (*p* < 0.05 vs. baseline/sham), LVEF decreased (*p* < 0.05 vs. sham/sh‐NC), LVFS decreased (*p* < 0.05 vs. baseline/sham); at 8 weeks, LVEDD/LVESD reduced and LVEF/LVFS improved (*p* < 0.05 vs. baseline, sham, sh‐NC, except LVFS vs. sham *p* > 0.05). Gambogic Amide reversed: decreased LVEF/LVFS at 4/8 weeks, increased LVESD at 8 weeks vs. sh‐RBM25 (*p* < 0.05; *p* < 0.05 vs. baseline/sham).

### 3.5 RBM25 Elevated NT‐Pro BNP, CRP, IL‐6, and TNF‐α Levels via the MAPK Pathway in Heart Failure

**FIGURE 4:**
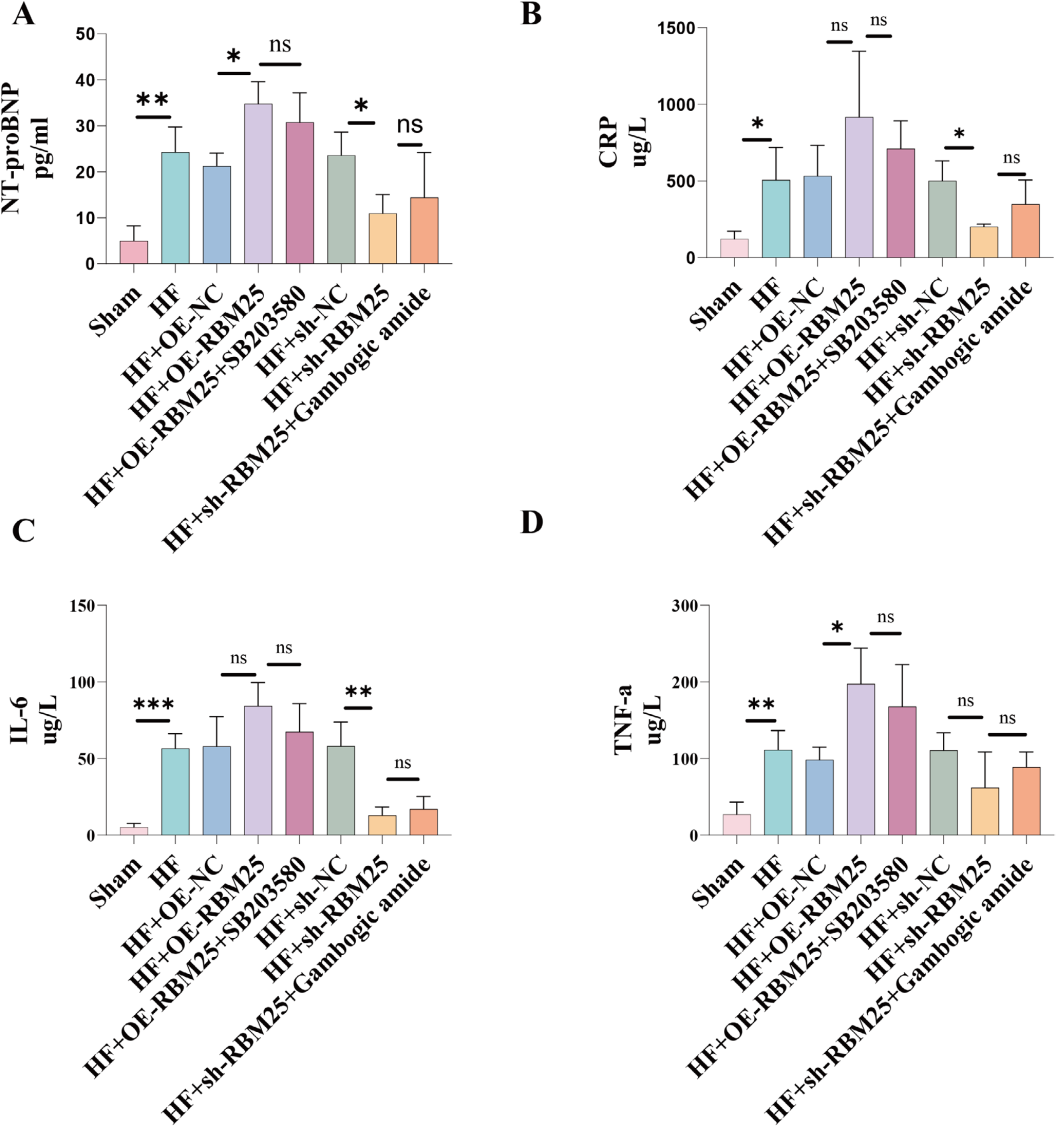
ELISA analysis of NT‐pro BNP (A), CRP (B), IL‐6 (C), and TNF‐α (D) levels in the serum of rats from 8 groups. *p < 0.05, **p < 0.01, ***p < 0.001, ns, non‐significant.

To investigate whether RBM25 exacerbates HF by positively regulating the MAPK signaling pathway, HF severity indicator NT‐pro BNP, and cardiomyocyte fibrosis and inflammation indicators CRP, IL‐6, and TNF‐α were measured using ELISA (Figure 4). Compared with the sham group, the HF group exhibited significantly elevated levels of NT‐pro BNP, CRP, IL‐6, and TNF‐α (*p* < 0.05), suggesting that HF conditions are associated with the upregulation of these markers. Further analysis revealed that the overexpression of RBM25 significantly increased NT‐pro BNP and TNF‐α levels (*p* < 0.05), while CRP and IL‐6 levels showed no significant difference compared to the HF group (*p* > 0.05). In contrast, RBM25 inhibition significantly decreased NT‐pro BNP, CRP, and IL‐6 levels (*p* < 0.05), while TNF‐α levels were not significantly altered (*p* > 0.05).

To explore the potential role of the MAPK signaling pathway in RBM25‐mediated regulation, SB203580 and Gambogic Amide were modulated in the context of RBM25 overexpression or suppression. In the RBM25 overexpression group, the addition of a selective p38 MAPK inhibitor did not significantly alter NT‐pro BNP, CRP, IL‐6, and TNF‐α levels (*p* > 0.05). In the sh‐RBM25 group, the introduction of a p38 MAPK activator did not significantly change these markers (*p* > 0.05).

### 3.6 RBM25 Promotes Heart Failure by Activating Cardiomyocyte Apoptosis Through Positive Regulation of the p38 MAPK Signaling Pathway

**FIGURE 5:**
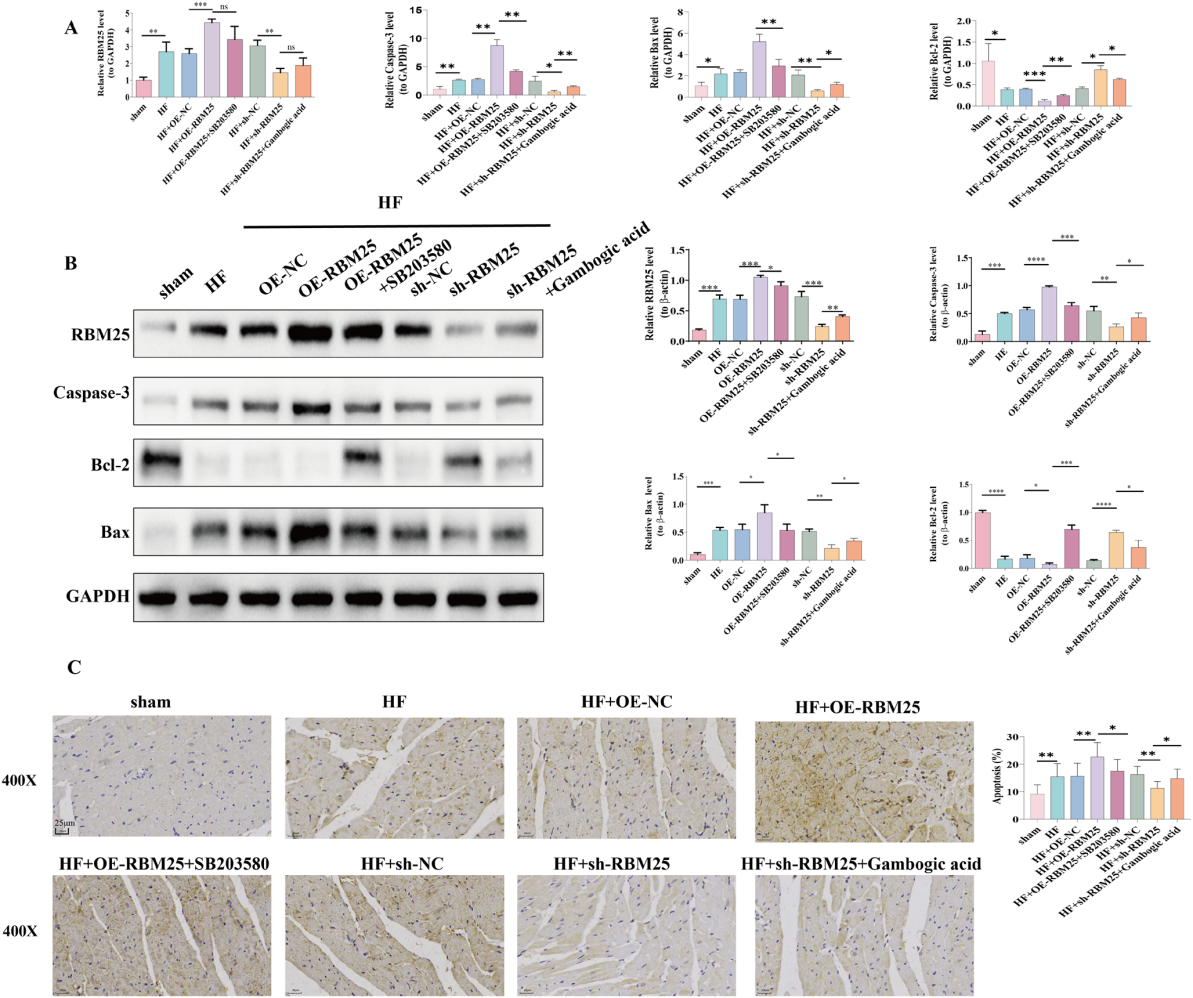
The level of apoptotic genes Caspase‐3, Bax, and Bcl‐2 expression detected by (A) qPCR analysis; (B) Western blotting analysis; (C) TUNEL staining to assess apoptosis rate in tissue samples, magnification 400X, scale bar 25 μm. *p < 0.05, **p < 0.01, ***p < 0.001, ns, non‐significant.

**FIGURE 6:**
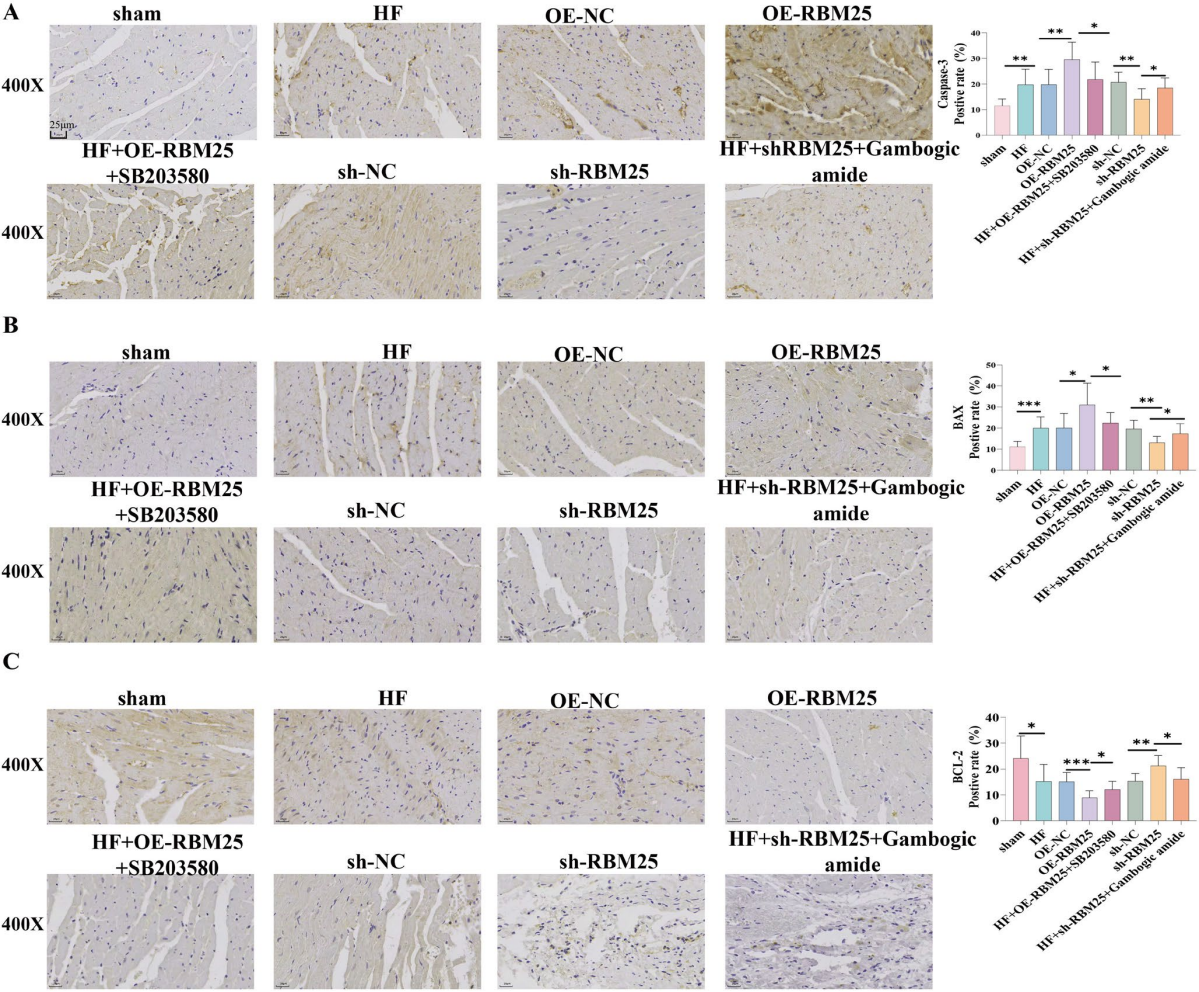
IHC analysis of RBM25 and apoptotic genes Caspase‐3, Bax, and Bcl‐2 expression. Magnification 400X, scale bar 25 μm.*p < 0.05, **p < 0.01, ***p < 0.001, ns, non‐significant.

To investigate whether RBM25 aggravates HF by promoting cardiomyocyte apoptosis through positive regulation of the p38 MAPK signaling pathway, we analyzed the expression levels of RBM25, pro‐apoptotic genes (Caspase‐3 and Bax), and the anti‐apoptotic gene (Bcl‐2) in myocardial tissues from eight groups of rats. Quantitative PCR (qPCR; Figure 5A) and Western blot (WB; Figure 5B) were used to measure mRNA and protein levels, respectively. Additionally, TUNEL staining (Figure 5C) was employed to quantitatively assess apoptosis severity, while IHC (Figure 6) was utilized to evaluate protein localization and expression.

Compared with the control group, the HF group displayed significantly higher mRNA and protein levels of RBM25, Caspase‐3, and Bax (*p* < 0.05), along with a notable decrease in Bcl‐2 expression (Figure 5A,B, *p* < 0.05). The apoptosis rate, as presented by Figure 5C, was also significantly elevated in the HF group (*p* < 0.05). In the RBM25 overexpression group, Caspase‐3 and Bax expression levels were markedly upregulated (*p* < 0.05), while Bcl‐2 expression was significantly downregulated (*p* < 0.05), resulting in a substantial increase in apoptosis ratio (*p* < 0.05). Trends in other markers did not reach significance (*p* > 0.05).

Further analysis revealed that the introduction of a p38 MAPK signaling inhibitor in the RBM25 overexpression group reversed these effects: Caspase‐3 and Bax expression levels were significantly reduced (*p* < 0.05), while the Bcl‐2 expression was significantly increased (*p* < 0.05), and the apoptosis rate was substantially diminished (*p* < 0.05). Conversely, adding a MAPK activator in the HF + sh‐RBM25 group restored Caspase‐3 and Bax expression to higher levels (*p* < 0.05), suppressed Bcl‐2 expression (*p* < 0.05), and significantly elevated the apoptosis rate (*p* < 0.05).

Additionally, immunohistochemistry (IHC) was employed to assess the expression of Caspase‐3, Bax, and the anti‐apoptotic marker Bcl‐2, with findings consistent with those presented in Figure 5A,B. Collectively, these results demonstrate that RBM25 exacerbates HF by enhancing the expression of pro‐apoptotic genes and suppressing anti‐apoptotic gene expression through positive regulation of the p38 MAPK signaling pathway.

### 3.7 RBM25 Regulates the Expression of MAP4K4, ERK, C‐FOS, EGR1, PARP1 to Promote Heart Failure

**FIGURE 7:**
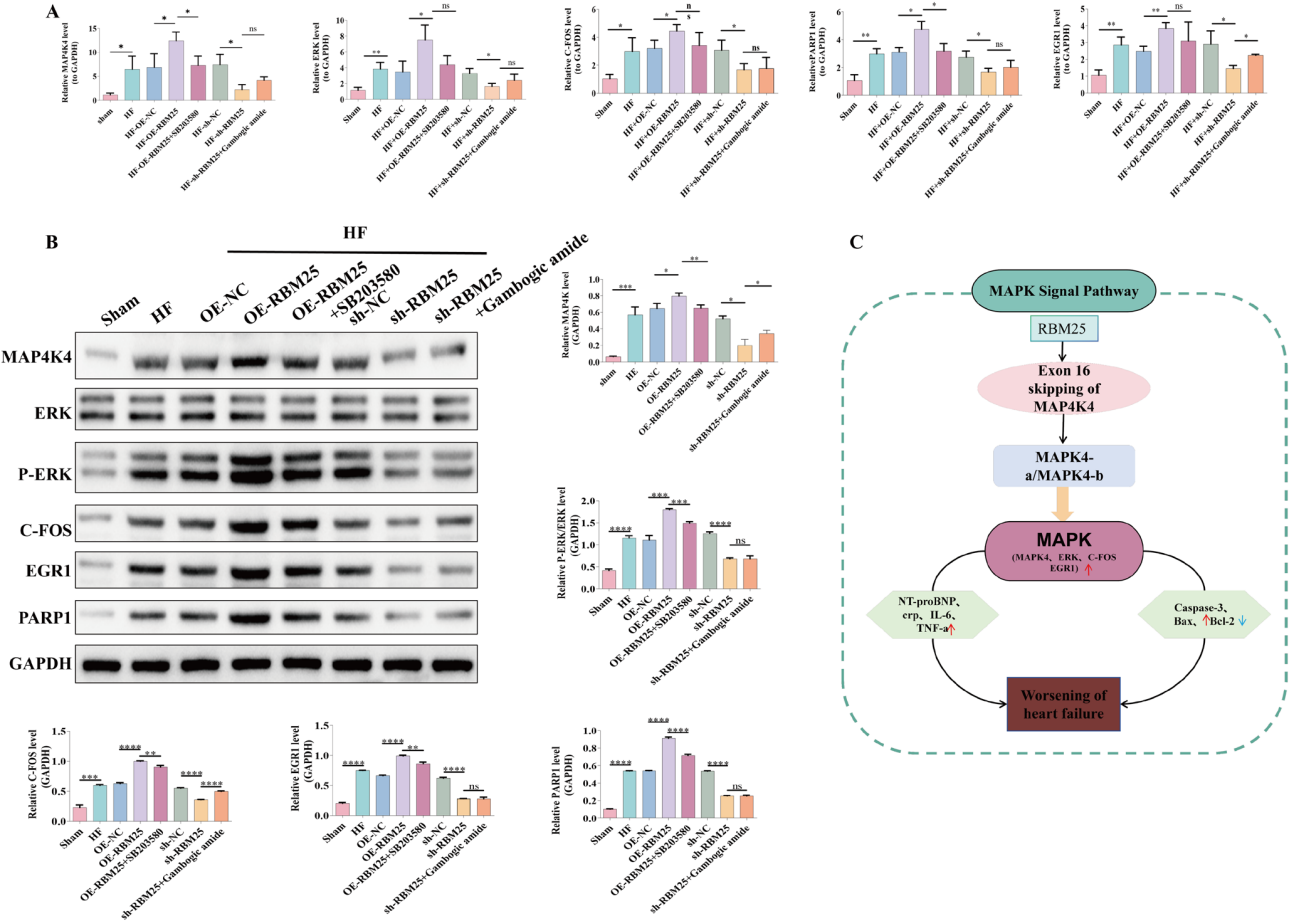
RBM25 regulates the expression of MAP4K4, ERK, C‐FOS, EGR1, and PARP1 via the MAPK pathway and its involvement in post‐myocardial infarction apoptosis. (A) qPCR analysis of MAP4K4, ERK, C‐FOS, EGR1, and PARP1. (B) Western blot analysis of MAPK pathway proteins: MAP4K4, p‐ERK, P‐ERK/ERK ratio, C‐FOS, EGR1, and PARP1. (C) Schematic of the study's hypothesis. Statistical significance: Ns *p* > 0.05, *p < 0.05, **p < 0.01, ***p < 0.001, ****p < 0.0001.

To further investigate the key target genes that the p38 MAPK signaling pathway is involved in under the regulation of RBM25, including MAP4K4, ERK, C‐FOS, EGR1, and PARP1, we analyzed their expression levels in myocardial tissues from eight experimental groups using qPCR, WB (Figure 7). Compared to the sham group, the HF group exhibited significant upregulation of MAP4K4, ERK, P‐ERK, C‐FOS, EGR1, and PARP1 at the mRNA level (*p* < 0.05). Corresponding protein levels of MAP4K4, P‐ERK, C‐FOS, EGR1, and PARP1 were also significantly elevated (*p* < 0.05), whereas the total protein levels of ERK showed no significant change. These elevations in total MAP4K4 protein levels align with the RBM25‐mediated exon 16 skipping observed in qPCR analyses.

RBM25 overexpression further amplified these effects, significantly increasing the mRNA and protein levels of MAP4K4, ERK, C‐FOS, EGR1, and PARP1 (*p* < 0.05). Compared to the RBM25 overexpression group, p38 MAPK pathway inhibition significantly reduced the mRNA and protein levels of MAP4K4, P‐ERK, C‐FOS, EGR1, and PARP1 (*p* < 0.05), as consistently confirmed by qPCR and WB analyses. Conversely, under RBM25 inhibition conditions, the addition of an indirect activation of the MAPK pathway partially restored MAPK pathway activity. The mRNA levels of EGR1 were significantly elevated (*p* < 0.05), and protein levels of MAP4K4 and C‐FOS were also restored to significant levels (*p* < 0.05).

## 4 Discussion

In this study, a HF model was established in SD rats via LAD coronary artery ligation to explore the role of RBM25 in myocardial infarction and HF. Through lentiviral‐mediated overexpression and suppression of RBM25, as well as computational modeling, it was demonstrated that overexpression of RBM25 leads to exon 16 skipping in MAP4K4, generating a variant with enhanced binding to MAP3K1, thereby activating the p38 MAPK pathway. Overexpression of RBM25 significantly upregulated key MAPK‐related genes and proteins, including MAP4K4, P‐ERK, C‐FOS, EGR1, and PARP1, as validated by ELISA, qPCR, WB, and IHC analyses. These findings establish RBM25 as a pivotal regulator of MAPK‐driven myocardial injury.

This study delved deeper into the mechanisms through which RBM25 influences HF, with a particular focus on the roles of p38 MAPK inhibition. The findings revealed that the p38 MAPK inhibitor SB203580 markedly reduced the myocardial infarction area caused by RBM25 overexpression. Importantly, introducing a p38 MAPK inhibitor in the RBM25 overexpression group reversed these changes, as evidenced by significant reductions in the expression levels of Caspase‐3 and Bax, an increase in Bcl‐2 expression, and a substantial decrease in the apoptosis rate. These findings align with existing evidence implicating excessive activation of the p38 MAPK signaling pathway in cardiomyocyte apoptosis. For example, activation of the NOX‐ROS‐MAPK axis under systemic inflammatory conditions leads to increased ROS levels, which in turn drive the phosphorylation of JNK and p38 subfamilies, thereby promoting apoptosis [24]. Similarly, the TAK1‐JNK/p38 MAPK pathway has been shown to mediate cardiomyocyte apoptosis and pathological cardiac remodeling under pressure overload stress [25]. The link from p38 MAPK activation to these apoptotic outcomes involves immediate downstream targets such as transcription factors ATF2 and MEF2C, which phosphorylate pro‐apoptotic regulators like Bad and Bim, triggering mitochondrial cytochrome c release and caspase‐9/3 activation, ultimately exacerbating HF remodeling [26, 27].

Together, these results underscore the pivotal role of p38 MAPK‐dependent apoptotic pathways in RBM25‐induced myocardial injury and HF progression. Activation of the MAPK pathway via the TrkA agonist Gambogic Amide mitigated the protective effects of RBM25 silencing, in the HF + sh‐RBM25 group restored elevated levels of Caspase‐3 and Bax, suppressed Bcl‐2 expression, and significantly heightened the apoptosis rate, likely by reactivating downstream pro‐apoptotic signaling cascades such as mitochondrial dysfunction and PARP1 cleavage. In RBM25‐overexpressing models, p38 MAPK inhibition with SB203580 did not result in statistically significant changes in HF biomarkers (NT‐proBNP, CRP, IL‐6, and TNF‐α; *p* > 0.05), which may reflect the study's limited statistical power due to small sample size. Although prior literature indicates p38 MAPK can modulate inflammatory cascades through NF‐κB regulation or direct cytokine expression [28].

Building on the established importance of RBPs in alternative splicing and cardiovascular disease, as well as our prior research in this field, this study further elucidates the regulatory role of RBM25 in HF. Specifically, our findings demonstrate that RBM25 mediates the alternative splicing of MAP4K4, resulting in exon 16 skipping and the generation of the MAP4K4‐b transcript. While the precise mechanism by which RBM25 specifically targets exon 16 of MAP4K4 remains to be fully elucidated, its RRM and SR domains likely enable sequence‐specific binding to cis‐regulatory elements in the pre‐mRNA, such as exonic splicing silencers (ESS) or intronic splicing enhancers (ISE), promoting exon skipping. RBM25 has been shown to act as a global splicing factor that predominantly favors exon inclusion but can also facilitate skipping in context‐dependent manners, potentially through interactions with spliceosome components or competing splicing factors [29, 30]. In analogous systems, splicing cascades involving RNA‐binding proteins like RBM4a regulate MAP4K4 exon inclusion via SRSF3, influencing MAPK‐mediated cellular processes [31], suggesting that RBM25 may similarly modulate MAP4K4 splicing by recognizing purine‐rich motifs or G‐quadruplex structures near exon 16 junctions. Previous studies have shown that MAP4K4 binds to MAP3K1 (MEKK1) via its CNH domain [32]. Using AlphaFold3, we predicted that this splice variant induces a structural rearrangement in the kinase domain, potentially enhancing the CNH domain's binding affinity to MAP3K1 through allosteric effects. Subsequent molecular docking with HDOCK confirmed a significantly enhanced binding affinity between the variant and MAP3K1, may leading to increased activation of the MAPK signaling pathway. Given the established involvement of MAPK signaling in pathological processes such as cell proliferation, inflammation, and fibrosis mentioned before, the splicing regulation observed here may represent a critical mechanism underlying HF progression.

Insights from related splicing cascades in non‐cardiac models reinforce the potential role of MAP4K4 exon 16 skipping in modulating MAPK signaling. For instance, the RBM4a‐SRSF3‐MAP4K4 axis in adipogenesis and cancer demonstrates how splicing regulators generate exon‐specific isoforms that differentially activate JNK/MAPK pathways, contributing to pro‐apoptotic or migratory effects [31, 33]. In cardiomyocytes, MAP4K4 isoforms exhibiting linker region variations, including those involving exon 16, interact with STRIPAK complexes to regulate stress responses, which may lead to MAPK hyperactivation under pathological conditions [34]. These observations are consistent with our computational predictions using AlphaFold3 and HDOCK, which indicate enhanced MAP3K1 binding affinity for the ΔEx16 isoform and suggest a gain‐of‐function mechanism in signal transduction. Additionally, single‐cell analyses illustrate dynamic splicing events in kinase domains such as exon 16, thereby influencing pathway diversity across tissues [35]. The broader involvement of MAP4K4 in metabolic and cardiovascular stress responses, including vascular inflammation, further highlights the likely contributions of its isoforms to HF progression [36, 37]. Complementing these external findings, our Pearson correlation analyses show associations between ΔEx16 ratio and downstream effectors.

Our results align closely with prior studies highlighting the pivotal role of RBPs and alternative splicing in cardiac pathology. Hoogenhof et al. comprehensively reviewed the essential contributions of RNA splicing to cardiac development and disease processes [10], noting that aberrant exon skipping in Titin, as observed in individuals with dilated cardiomyopathy (DCM), results in the production of a larger N2BA‐G isoform that compromises myocardial contractility and accelerates HF progression. Similarly, the alternative splicing of calcium‐regulating genes such as CamkIIδ and Ryr2 has been shown to disrupt calcium homeostasis, significantly increasing the risk of arrhythmias and sudden cardiac death [10, 38]. Moreover, deficiencies in splicing factors such as SRSF1 and hnRNP U exacerbate splicing errors in genes like CamkIIδ, further contributing to contractile dysfunction and DCM development [10]. Li et al. [39] provided additional evidence for the extensive prevalence of splicing abnormalities in HF through RNA sequencing of myocardial tissue, identifying widespread exon‐skipping events and selective 3′ splice site usage. Their pathway enrichment analysis linked these splicing anomalies to dysregulated pathways involved in PPAR signaling, actin cytoskeleton regulation, and muscle contraction, and implicated key splicing factors such as RBM5, ZRANB2, hnRNPF, and hnRNPA0. These findings collectively underscore the importance of exon skipping as both a marker and a driver of HF pathology. In comparison to the sham‐operated group, the HF group exhibited significant upregulation of MAP4K4, ERK, P‐ERK, C‐FOS, EGR1, and PARP1, indicating pronounced activation of the p38 MAPK pathway and increased apoptotic activity. RBM25 overexpression further enhanced MAP4K4 expression and ERK phosphorylation, accompanied by upregulation of downstream targets such as C‐FOS, EGR1, and elevated PARP1 levels, the latter playing a key role in DNA damage repair by promoting single‐strand break repair (SSBR) failure, leading to the accumulation of double‐strand breaks (DSBs) and triggering apoptosis [40].

The p38 MAPK inhibitor SB203580 significantly reduced MAP4K4 and PARP1 mRNA levels, suppressing transcription and apoptotic signaling. While ERK, C‐FOS, and EGR1 mRNA levels showed no statistically significant changes (*p* > 0.05), possibly due to multiple MAPK branches and limited sample size. At the protein level, however, SB203580 markedly decreased ERK, P‐ERK, C‐FOS, EGR1, and PARP1 expression, confirming its role in blocking MAPK activation and apoptosis. The pro‐apoptotic effects of RBM25 primarily involve the MAP4K4‐p38 MAPK branch, supported by SB203580's specific inhibition and MAP4K4's regulation of p38 via Src and CXCR2‐mediated signaling [41]. Although our assays primarily focused on ERK phosphorylation as a downstream readout of MAPK signaling, SB203580's specificity as a p38 MAPK inhibitor suggests that its effects may involve indirect modulation of ERK through known crosstalk within the MAPK cascades. In cardiac models, p38 activation has been shown to influence ERK signaling, often via shared regulatory mechanisms such as protein phosphatase 2A (PP2A), leading to coordinated responses in apoptosis and hypertrophy [42]. This crosstalk supports our interpretation of p38‐mediated attenuation of RBM25‐induced myocardial injury, as observed in reduced infarct size and apoptosis markers. However, the absence of direct p38 measurements in our study introduces some ambiguity, and future investigations should assess p38 phosphorylation levels alongside broader MAPK inhibitors to confirm pathway specificity and disentangle these interactions.

In the RBM25‐inhibition model, treatment with Gambogic Amide—a selective TrkA agonist that activates the MAPK signaling pathway [43]—partially restored the expression of MAP4K4 and downstream effectors (e.g., C‐FOS and EGR1). This observation provides mechanistic evidence for reactivation of the p38 MAPK signaling cascade. Concurrently, this intervention was associated with a significant increase in myocardial infarct size and cardiomyocyte apoptosis rates, effectively abrogating the protective effects conferred by RBM25 silencing alone. Although Gambogic Amide's activation of MAPK occurs indirectly via TrkA receptor stimulation, leading to tyrosine phosphorylation and downstream ERK/MAPK signaling [43], it has been shown to reduce infarct volume in neurodegeneration models, supporting its utility in validating pathway reactivation despite potential off‐target effects [44]. These findings demonstrate that indirect activation of the MAPK pathway overrides the beneficial effects of RBM25 knockdown, thereby underscoring the pivotal role of p38 MAPK signaling in mediating the pathological effects of RBM25 during HF progression. However, the indirect nature of Gambogic Amide may involve other pathways, and future studies should employ more direct MAPK activators to refine this mechanism.

This study is among the first to identify RBM25 as a regulator of ischemic HF through its modulation of MAP4K4 alternative splicing and downstream activation of the p38 MAPK signaling pathway using a post‐infarction rat model. The findings highlight RBM25 as a promising therapeutic target, with technologies such as CRISPR‐Cas9 and antisense oligonucleotides offering potential for precise interventions [45, 46, 47].

However, several limitations should be acknowledged. The LAD ligation‐induced ischemic HF rat model offers mechanistic insights but fails to replicate chronic, multifactorial human ischemic cardiomyopathy, with high perioperative mortality reducing sample size and statistical power in small groups. Species‐specific differences in splicing and signaling limit translational relevance (e.g., MAP4K4 exon 16 skipping may not occur in humans). Using young, healthy animals overlooks age/comorbidity effects in older patients. The short eight‐week follow‐up may miss long‐term remodeling, and pericardial lentiviral/pharmacological interventions (e.g., SB203580, Gambogic Amide) lack clinical translatability. Human tissue or iPSC‐cardiomyocyte validation is essential. The MAP4K4‐MAPK focus underexplores pathways like JNK/p38 (SB203580's p38 specificity), PI3K/AKT, or NF‐κB. Mechanistic gaps include missing RBM25‐MAP4K4 pre‐mRNA binding evidence (e.g., RIP‐qPCR) or ΔEx16 isoform necessity (e.g., rescue experiments, Western blot). Whole‐tissue analyses ignore cellular heterogeneity, non‐significant trends (e.g., ELISA/IHC) indicate larger samples needed, and Gambogic Amide risks off‐target effects. Despite these limitations, this study establishes RBM25 as a pivotal player in HF progression and provides a foundation for developing targeted therapies to address this complex condition.

## 5 Conclusion

RBM25 may exacerbate HF by partially activating the p38 MAPK signaling pathway through promotion of exon 16 skipping in MAP4K4 at the transcript level, which possibly results in increased cardiomyocyte apoptosis and myocardial injury. Targeting RBM25 may offer a promising therapeutic strategy for mitigating HF progression.

## Author Contributions

Hao Li analyzed the data, prepared figures and/or tables, wrote drafts of the manuscript, and approved the final version. Keyi Zhang contributed to data collection and analysis. Chen Liu performed imaging studies and contributed to data interpretation. Xin Tian conducted statistical analyses and contributed to manuscript preparation. Guangli Zhou provided technical support and contributed to experimental protocols. Wanshu Liu and Lingmin Zhao participated in data curation and manuscript editing. Luqiao Wang provided clinical expertise and contributed to data interpretation. Ping Yang supervised the study, designed and conducted the experiments, reviewed and revised the manuscript, and approved the final version as the corresponding author.

## Funding

This work was supported by the National Natural Science Foundation of China under grant (82360082) and (81760074); the Special Foundation Projects of Joint Applied Basic Research of Yunnan Provincial Department of Science and Technology with Kunming Medical University under grant (202201AY070001‐064); the Foundation Projects of young and middle‐aged academic and technical leaders reserve talent of Yunnan province under grant (202305AC160048); the Yunnan Health Training Project of High‐Level Talents under grant (D‐2018020); Yunnan Province Metabolism‐related Cardiovascular Disease Innovation Team, grant number: 202405AS350014; the funders had no role in study design, data collection and analysis, decision to publish, or preparation of the manuscript.

## Ethics Statement

All animal experiments were conducted in strict accordance with the 3R principles (replacement, reduction, and refinement) for the care and use of laboratory animals. Experimental procedures were approved by the Animal Experiment Ethics Review Committee of Kunming Medical University (approval number: kmmu20211238) (File S1).

## Consent

The authors have nothing to report.

## Conflicts of Interest

The authors declare no conflicts of interest.

## Supporting information


**Figure S1:** Re‐analysis of RNA‐seq and iRIP‐seq data from RBM25‐overexpressed H9c2 cells. (A) Volcano plot showing the number of identified differentially expressed genes (DEGs), with red dots indicating significantly upregulated genes and blue dots indicating significantly downregulated genes. The criteria for significant differential expression were fold change (FC) ≥ 1.5 or ≤ 2/3, and *p* < 0.01. (B) Heatmap illustrating expression levels of genes with significant differential expression. (C) Bar chart of KEGG pathway enrichment for downregulated DEGs. (D) Bar chart of KEGG pathway enrichment for upregulated DEGs. (E) Bar chart of KEGG pathway enrichment for differentially alternatively spliced genes (RASGs). (F) Bar chart showing the number of identified significant differential alternative splicing events by type. (G) Sample correlation clustering analysis based on normalized RPKM (reads per kilobase of transcript per million mapped reads) values across samples. (H) KEGG pathway enrichment analysis results for the 31 overlapping genes between RBM25 binding peak genes and RASGs. (I) MAP4K4 exon skipping (ES) event, showing read distribution for transcripts in RBM25‐overexpressed (orange) and control (purple) samples, alongside the PSI (percent spliced in) ratios for experimental and control groups.

**Figure S2:** RBM25 overexpression/suppression lentivirus packaging and efficiency verification. (A) Packaging of RBM25 knockdown/overexpression lentivirus; (B) screening of the optimal RBM25 inhibition, with sh‐RBM25‐2 showing the best knockdown efficiency compared to the control group; (C) verification of RBM25 overexpression.

**Figure S3:** Pearson correlation analyses of RBM25 protein levels, MAP4K4 ΔEx16 ratio, and p‐ERK levels. Scatter plots showing Pearson correlations based on individual sample data from HF, OE‐NC, OE‐RBM25, sh‐NC, and sh‐RBM25 groups (*n* = 6 per group). All values are relative levels normalized to GAPDH. Left: Relative p‐ERK level (x‐axis) vs. relative RBM25 level (y‐axis; r = 0.8391, p < 0.0001). Middle: Relative p‐ERK level (x‐axis) vs. relative ΔEx16 ratio (y‐axis; r = 0.5928, p = 0.0008). Right: Relative RBM25 level (x‐axis) versus relative ΔEx16 ratio (y‐axis; r = 0.5890, p = 0.0008). Each point represents one sample.

**Table S1:** The primer sequences used in real‐time qPCR.

**Table S2:** Statistical analysis of molecular docking scores (docking score, kcal/mol).

**Table S3:** Changes in rat echocardiographic parameters.

**Data S1:** fba270074‐sup‐0007‐DataS1.

## Data Availability

Stored in repository. The datasets analyzed during the current study are available from the corresponding author on reasonable request.

## References

[1] G. B. D. Disease , I. Injury , and C. Prevalence , “Global, Regional, and National Incidence, Prevalence, and Years Lived With Disability for 354 Diseases and Injuries for 195 Countries and Territories, 1990–2017: A Systematic Analysis for the Global Burden of Disease Study 2017,” Lancet 392 (2018): 1789–1858, 10.1016/S0140-6736(18)32279-7. 30496104 PMC6227754

[2] A. Groenewegen , F. H. Rutten , A. Mosterd , and A. W. Hoes , “Epidemiology of Heart Failure,” European Journal of Heart Failure 22 (2020): 1342–1356, 10.1002/ejhf.1858. 32483830 PMC7540043

[3] C. W. Tsao , A. Lyass , D. Enserro , et al., “Temporal Trends in the Incidence of and Mortality Associated With Heart Failure With Preserved and Reduced Ejection Fraction,” JACC Heart Failure 6 (2018): 678–685, 10.1016/j.jchf.2018.03.006. 30007560 PMC6076350

[4] L. Schirone , M. Forte , L. D'Ambrosio , V. Valenti , and D. Vecchio , “An Overview of the Molecular Mechanisms Associated With Myocardial Ischemic Injury: State of the Art and Translational Perspectives,” Cells 11 (2022): 1165, 10.3390/cells11071165. 35406729 PMC8998015

[5] K. K. Khush , E. Hsich , L. Potena , J. Patel , and M. Sabatino , “The International Thoracic Organ Transplant Registry of the International Society for Heart and Lung Transplantation: Thirty‐Eighth Adult Heart Transplantation Report—2021; Focus on Recipient Characteristics,” Journal of Heart and Lung Transplantation 40 (2021): 1035–1049, 10.1016/j.healun.2021.07.015. PMC10282986 34419370

[6] D. Gong , F. Yang , and F. Li , “Crystal Structure and Functional Characterization of the Human RBM25 PWI Domain and Its Flanking Basic Region,” Biochemical Journal 450 (2013): 85–94, 10.1042/BJ20121382. 23190262 PMC3553564

[7] X. Huang , J. Liu , X. Mo , H. Liu , C. Wei , and L. Huang , “Systematic Profiling of Alternative Splicing Events and Splicing Factors in Left‐ and Right‐Sided Colon Cancer,” Aging (Albany N. Y.) 11 (2019): 8270–8293, 10.18632/aging.102319. PMC6814588 31586988

[8] L. Lin , J. Chu , S. An , X. Liu , and R. Tan , “The Biological Mechanisms and Clinical Roles of RNA‐Binding Proteins in Cardiovascular Diseases,” Biomolecules 14 (2024): 1056, 10.3390/biom14091056. 39334823 PMC11430443

[9] X. Tian , G. Zhou , H. Li , X. Zhang , L. Zhao , and K. Zhang , “RBM25 Binds to and Regulates Alternative Splicing Levels of Slc38a9, Csf1, and Coro6 to Affect Immune and Inflammatory Processes in H9c2 Cells,” PeerJ 11 (2023): e16312, 10.7717/peerj.16312. 37953772 PMC10637245

[10] M. M. G. van den Hoogenhof , Y. M. Pinto , and E. E. Creemers , “RNA Splicing: Regulation and Dysregulation in the Heart,” Circulation Research 118 (2016): 454–468, 10.1161/CIRCRESAHA.115.307872. 26846640

[11] J. K. Hahn , B. Neupane , K. Pradhan , Q. Zhou , Y. Chen , and Y. Sun , “The Assembly and Evaluation of Antisense Oligonucleotides Applied in Exon Skipping for Titin‐Based Mutations in Dilated Cardiomyopathy,” Journal of Molecular and Cellular Cardiology 131 (2019): 12–19, 10.1016/j.yjmcc.2019.04.014. 30998980

[12] R. Yu , Q. Li , Z. Feng , L. Cai , and Q. Xu , “m6A Reader YTHDF2 Regulates LPS‐Induced Inflammatory Response,” International Journal of Molecular Sciences 20 (2019): 1323, 10.3390/ijms20061323. 30875984 PMC6470741

[13] G. Olivetti , F. Quaini , R. Sala , and C. Lagrasta , “Acute Myocardial Infarction in Humans Is Associated With Activation of Programmed Myocyte Cell Death in the Surviving Portion of the Heart,” Journal of Molecular and Cellular Cardiology 28 (1996): 2005–2016, 10.1006/jmcc.1996.0197. 8899559

[14] G. Takemura , M. Ohno , Y. Hayakawa , J. Misao , and N. Hayakawa , “Role of Apoptosis in the Disappearance of Infiltrated and Proliferated Interstitial Cells After Myocardial Infarction,” Circulation Research 82 (1998): 1130–1138, 10.1161/01.RES.82.11.1130. 9633913

[15] D. P. Del Re , D. Amgalan , A. Linkermann , K. Hanggi , and W. Zheng , “Fundamental Mechanisms of Regulated Cell Death and Implications for Heart Disease,” Physiological Reviews 99 (2019): 1765–1817, 10.1152/physrev.00022.2018. 31364924 PMC6890986

[16] H. O. Alharbi , P. H. Sugden , and A. Clerk , “Mitogen‐Activated Protein Kinase Signalling in Rat Hearts During Postnatal Development: MAPKs, MAP3Ks, MAP4Ks and DUSPs,” Cellular Signalling 124 (2024): 111397, 10.1016/j.cellsig.2024.111397. 39251052

[17] J. Li , A. M. Salvador , G. Li , N. Valkov , and O. Ziegler , “Mir‐30d Regulates Cardiac Remodeling by Intracellular and Paracrine Signaling,” Circulation Research 128 (2020): e1–e23, 10.1161/CIRCRESAHA.120.317244. 33092465 PMC7790887

[18] Y. Hou , C. Huang , X. Cai , J. Zhao , and W. Zhang , “Improvements in the Establishment of a Rat Myocardial Infarction Model,” Journal of International Medical Research 39 (2011): 1284–1292, 10.1177/147323001103900416. 21986130

[19] J. Abramson , J. Adler , J. Dunger , et al., “Addendum: Accurate Structure Prediction of Biomolecular Interactions With AlphaFold 3,” Nature 636 (2024): E4, 10.1038/s41586-024-08416-7. 39604737 PMC11634763

[20] R. Anandakrishnan , B. Aguilar , and A. V. Onufriev , “H++ 3.0: Automating pK Prediction and the Preparation of Biomolecular Structures for Atomistic Molecular Modeling and Simulations,” Nucleic Acids Research 40 (2012): W537–W541, 10.1093/nar/gks375. 22570416 PMC3394296

[21] E. F. Pettersen , T. D. Goddard , C. C. Huang , et al., “UCSF Chimera—A Visualization System for Exploratory Research and Analysis,” Journal of Computational Chemistry 25 (2004): 1605–1612, 10.1002/jcc.20084. 15264254

[22] Y. Yan , D. Zhang , P. Zhou , B. Li , and S. Y. Huang , “HDOCK: A Web Server for Protein‐Protein and Protein‐DNA/RNA Docking Based on a Hybrid Strategy,” Nucleic Acids Research 45 (2017): W365–W373, 10.1093/nar/gkx407. 28521030 PMC5793843

[23] R. A. Laskowski and M. B. Swindells , “LigPlot+: Multiple Ligand‐Protein Interaction Diagrams for Drug Discovery,” Journal of Chemical Information and Modeling 51 (2011): 2778–2786, 10.1021/ci200227u. 21919503

[24] Y. Wen , R. Liu , N. Lin , H. Luo , and J. Tang , “NADPH Oxidase Hyperactivity Contributes to Cardiac Dysfunction and Apoptosis in Rats With Severe Experimental Pancreatitis Through ROS‐Mediated MAPK Signaling Pathway,” Oxidative Medicine and Cellular Longevity 2019 (2019): 1, 10.1155/2019/4578175. PMC6532283 31210840

[25] J. Li , C. Yan , Y. Wang , C. Chen , H. Yu , and D. Liu , “GCN5‐Mediated Regulation of Pathological Cardiac Hypertrophy via Activation of the TAK1‐JNK/p38 Signaling Pathway,” Cell Death & Disease 13 (2022): 421, 10.1038/s41419-022-04881-y. 35490166 PMC9056507

[26] T. Yokota and Y. Wang , “p38 MAP Kinases in the Heart,” Gene 575 (2016): 369–376, 10.1016/j.gene.2015.09.030. 26390817 PMC4860190

[27] M. S. Marber , B. Rose , and Y. Wang , “The p38 Mitogen‐Activated Protein Kinase Pathway—A Potential Target for Intervention in Infarction, Hypertrophy, and Heart Failure,” Journal of Molecular and Cellular Cardiology 51 (2011): 485–490, 10.1016/j.yjmcc.2010.10.021. 21062627 PMC3061241

[28] M. Fisk , P. R. Gajendragadkar , K. M. Mäki‐Petäjä , I. B. Wilkinson , and J. Cheriyan , “The Case for Inhibiting p38 Mitogen‐Activated Protein Kinase in Heart Failure,” Frontiers in Pharmacology 6 (2015): 102, 10.3389/fphar.2015.00102. 26029107 PMC4428223

[29] S. M. Carlson , C. M. Soulette , Z. Yang , J. E. Elias , A. N. Brooks , and O. Gozani , “RBM25 Is a Global Splicing Factor Promoting Inclusion of Alternatively Spliced Exons and Is Itself Regulated by Lysine Mono‐Methylation,” Journal of Biological Chemistry 292 (2017): 13381–13390, 10.1074/jbc.M117.784371. 28655759 PMC5555197

[30] N. H. Gehring , S. Lamprinaki , A. E. Kulozik , and M. W. Hentze , “Disassembly of Exon Junction Complexes by PYM,” Cell 137 (2009): 536–548, 10.1016/j.cell.2009.02.042. 19410547

[31] H.‐Y. Peng , Y.‐C. Liang , T.‐H. Tan , H.‐C. Chuang , Y.‐J. Lin , and J.‐C. Lin , “RBM4a‐SRSF3‐MAP4K4 Splicing Cascade Constitutes a Molecular Mechanism for Regulating Brown Adipogenesis,” International Journal of Molecular Sciences 19 (2018): 2646, 10.3390/ijms19092646. 30200638 PMC6163301

[32] J. C. Loftus , Z. Yang , J. Kloss , H. Dhruv , N. L. Tran , and D. L. Riggs , “A Novel Interaction Between Pyk2 and MAP4K4 Is Integrated With Glioma Cell Migration,” Journal of Signal Transduction 2013 (2013): 956580, 10.1155/2013/956580. 24163766 PMC3791834

[33] J.‐C. Lin , Y.‐C. Lee , T.‐H. Tan , et al., “RBM4‐SRSF3‐MAP4K4 Splicing Cascade Modulates the Metastatic Signature of Colorectal Cancer Cell,” Biochimica et Biophysica Acta, Molecular Cell Research 1865 (2018): 259–272, 10.1016/j.bbamcr.2017.11.005. 29138007

[34] S. J. Fuller , N. S. Edmunds , L. J. McGuffin , A. Narayanapanicker , and P. H. Sugden , “MAP4K4 Expression in Cardiomyocytes: Multiple Isoforms, Multiple Phosphorylations and Interactions With Striatins,” Biochemical Journal 478 (2021): 2121–2143, 10.1042/BCJ20210003. 34032269 PMC8203206

[35] Y. Song , O. B. Botvinnik , M. T. Lovci , et al., “Single‐Cell Alternative Splicing Analysis With Expedition Reveals Splicing Dynamics During Neuron Differentiation,” Molecular Cell 67 (2017): 148–161.e145, 10.1016/j.molcel.2017.06.003. 28673540 PMC5540791

[36] J. V. Virbasius and M. P. Czech , “Map4k4 Signaling Nodes in Metabolic and Cardiovascular Diseases,” Trends in Endocrinology and Metabolism 27 (2016): 465–475, 10.1016/j.tem.2016.04.006. PMC4912878 27160798

[37] R. J. Roth Flach , A. Skoura , A. Matevossian , et al., “Endothelial Protein Kinase MAP4K4 Promotes Vascular Inflammation and Atherosclerosis,” Nature Communications 6 (2015): 8995, 10.1038/ncomms9995. PMC4703891 26688060

[38] P. Ortiz‐Sánchez , M. Villalba‐Orero , M. M. López‐Olañeta , et al., “Loss of SRSF3 in Cardiomyocytes Leads to Decapping of Contraction‐Related mRNAs and Severe Systolic Dysfunction,” Circulation Research 125 (2019): 170–183, 10.1161/CIRCRESAHA.118.314515. 31145021 PMC6615931

[39] J. Li , D. Tu , S. Li , Z. Guo , and X. Song , “Identification of Alternative Splicing Regulatory Patterns and Characteristic Splicing Factors in Heart Failure Using RNA‐Seq Data and Machine Learning,” Heliyon 10 (2024): e35408, 10.1016/j.heliyon.2024.e35408. 39170450 PMC11336631

[40] C. Sun , Y. Fang , J. Yin , J. Chen , Z. Ju , and D. Zhang , “Rational Combination Therapy With PARP and MEK Inhibitors Capitalizes on Therapeutic Liabilities in RAS Mutant Cancers,” Science Translational Medicine 9 (2017), 10.1126/scitranslmed.aal5148. PMC5919217 28566428

[41] S. He , T. Hou , J. Zhou , et al., “Implication of CXCR2‐Src Axis in the Angiogenic and Osteogenic Effects of FP‐TEB,” npj Regenerative Medicine 24 (2024), 10.1038/s41536-024-00364-0. PMC11415383 39304660

[42] Q. Liu and P. A. Hofmann , “Protein Phosphatase 2A‐Mediated Cross‐Talk Between p38 MAPK and ERK in Apoptosis of Cardiac Myocytes,” American Journal of Physiology. Heart and Circulatory Physiology 286 (2004): H2204–H2212, 10.1152/ajpheart.01050.2003. 14962831

[43] S. Jang , M. Okada , I. Sayeed , et al., “Gambogic Amide, a Selective Agonist for TrkA Receptor That Possesses Robust Neurotrophic Activity, Prevents Neuronal Cell Death,” Proceedings of the National Academy of Sciences of the United States of America 104 (2007): 16329–16334, 10.1073/pnas.0706662104. 17911251 PMC2042206

[44] T. S. Thompson , A. Sefiani , and K. Burgess , “Small‐Molecule Trk Agonists: Where Do We Go From Here?,” Journal of Medicinal Chemistry 68 (2025): 15233–15259, 10.1021/acs.jmedchem.4c02365. 40679347 PMC12362621

[45] H. Maatz , M. Jens , M. Liss , et al., “RNA‐Binding Protein RBM20 Represses Splicing to Orchestrate Cardiac Pre‐mRNA Processing,” Journal of Clinical Investigation 124 (2014): 3419–3430, 10.1172/JCI74523. 24960161 PMC4109538

[46] T. Nishiyama , Y. Zhang , M. Cui , H. Li , S. Jiang , and W. Ma , “Precise Genomic Editing of Pathogenic Mutations in RBM20 Rescues Dilated Cardiomyopathy,” Science Translational Medicine 14 (2022): eade1633, 10.1126/scitranslmed.ade1633. 36417486 PMC10088465

[47] K. Y. Lee , E. M. McNally , and L. Mestroni , “Antisense Oligonucleotides Targeting RNA‐Binding Protein RBM20 Reduce Cardiac Fibrosis in a Mouse Model of Heart Failure,” Circulation Research 118 (2016): 847–856, 10.1161/circresaha.116.309396.

