# Supplementary figures and images for: RBM25 Regulates p38 MAPK Pathway Activation via Exon 16 Skipping of MAP4K4 in a Rat Model of Post‐Infarction Heart Failure
Source: FASEB Bioadv. 2025 Dec 16;7(12):e70074. doi: 10.1096/fba.2025-00201 (PMC12707302; doi:10.1096/fba.2025-00201)

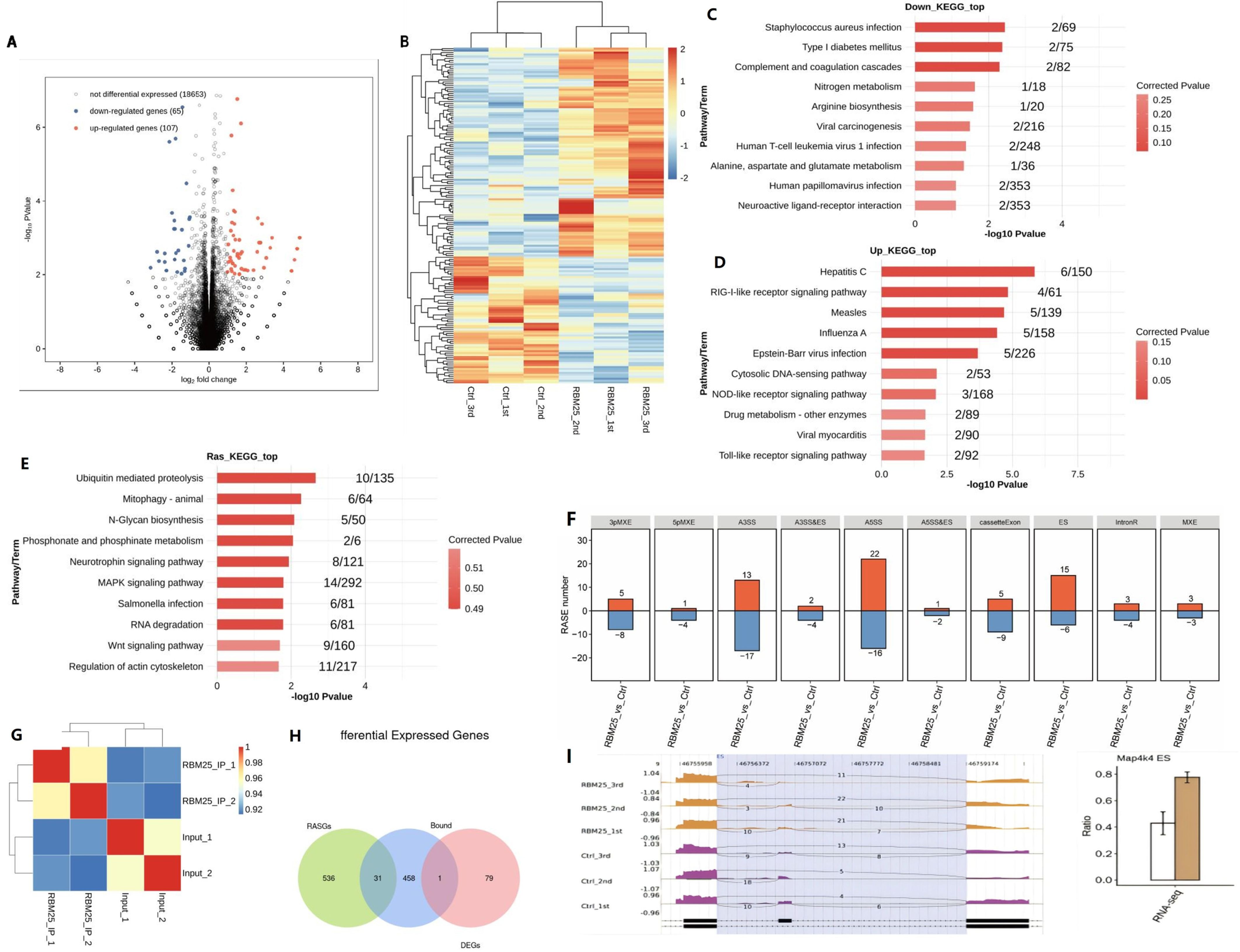

Supplement: Supplementary file 1 — Figure S1: Re‐analysis of RNA‐seq and iRIP‐seq data from RBM25‐overexpressed H9c2 cells. (A) Volcano plot showing the number of identified differentially expressed genes (DEGs), with red dots indicating significantly upregulated genes and blue dots indicating significantly downregulated genes. The criteria for significant differential expression were fold change (FC) ≥ 1.5 or ≤ 2/3, and p < 0.01. (B) Heatmap illustrating expression levels of genes with significant differential expression. (C) Bar chart of KEGG pathway enrichment for downregulated DEGs. (D) Bar chart of KEGG pathway enrichment for upregulated DEGs. (E) Bar chart of KEGG pathway enrichment for differentially alternatively spliced genes (RASGs). (F) Bar chart showing the number of identified significant differential alternative splicing events by type. (G) Sample correlation clustering analysis based on normalized RPKM (reads per kilobase of transcript per million mapped reads) values across samples. (H) KEGG pathway enrichment analysis results for the 31 overlapping genes between RBM25 binding peak genes and RASGs. (I) MAP4K4 exon skipping (ES) event, showing read distribution for transcripts in RBM25‐overexpressed (orange) and control (purple) samples, alongside the PSI (percent spliced in) ratios for experimental and control groups. [file FBA2-7-e70074-s004.jpg]

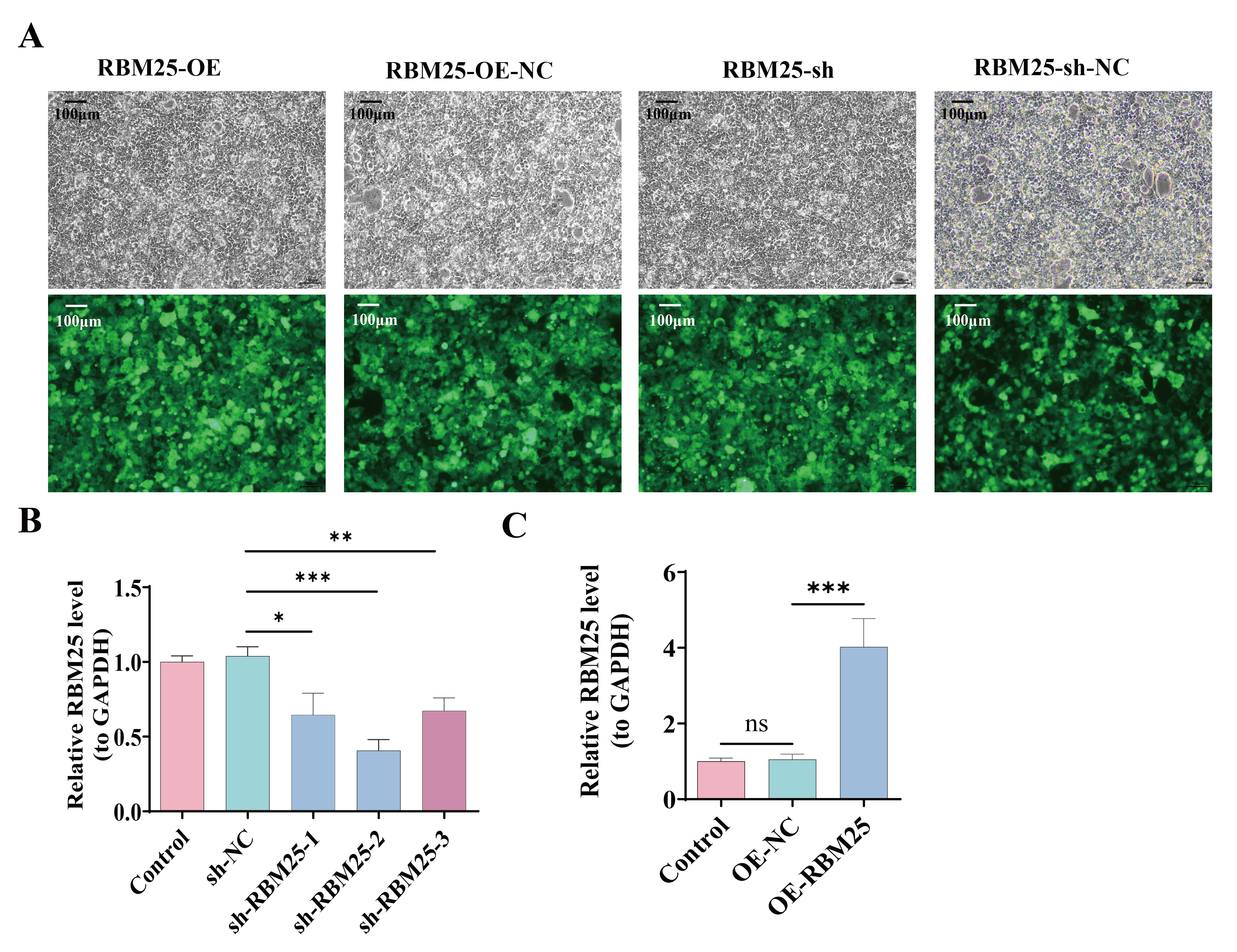

Supplement: Supplementary file 2 — Figure S2: RBM25 overexpression/suppression lentivirus packaging and efficiency verification. (A) Packaging of RBM25 knockdown/overexpression lentivirus; (B) screening of the optimal RBM25 inhibition, with sh‐RBM25‐2 showing the best knockdown efficiency compared to the control group; (C) verification of RBM25 overexpression. [file FBA2-7-e70074-s007.tif]

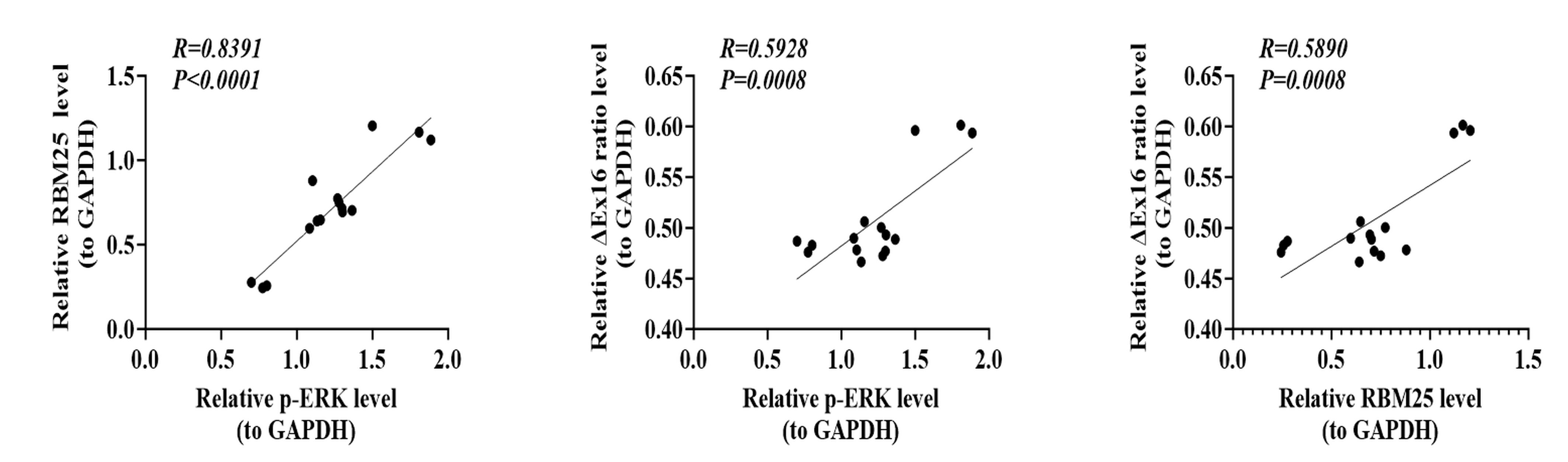

Supplement: Supplementary file 3 — Figure S3: Pearson correlation analyses of RBM25 protein levels, MAP4K4 ΔEx16 ratio, and p‐ERK levels. Scatter plots showing Pearson correlations based on individual sample data from HF, OE‐NC, OE‐RBM25, sh‐NC, and sh‐RBM25 groups (n = 6 per group). All values are relative levels normalized to GAPDH. Left: Relative p‐ERK level (x‐axis) vs. relative RBM25 level (y‐axis; r = 0.8391, p < 0.0001). Middle: Relative p‐ERK level (x‐axis) vs. relative ΔEx16 ratio (y‐axis; r = 0.5928, p = 0.0008). Right: Relative RBM25 level (x‐axis) versus relative ΔEx16 ratio (y‐axis; r = 0.5890, p = 0.0008). Each point represents one sample. [file FBA2-7-e70074-s001.jpg]
