# Supplementary material for: RBM25 Regulates p38 MAPK Pathway Activation via Exon 16 Skipping of MAP4K4 in a Rat Model of Post‐Infarction Heart Failure
Source: FASEB Bioadv. 2025 Dec 16;7(12):e70074. doi: 10.1096/fba.2025-00201 (PMC12707302; doi:10.1096/fba.2025-00201)
Supplement: Supplementary file 4 — Table S1: The primer sequences used in real‐time qPCR. [file FBA2-7-e70074-s003.docx]

Table S1 The primer sequences used in real-time qPCR.

| Gene | sequence（5‘-3’） |
| --- | --- |
| R-GAPDH F | GACCCCTTCATTGACCTCAAC |
| R-GAPDH R | GCCATCACGCCACAGCTTTCC |
| RBM25 F | TTTTCTTAGCGGCGGACTGG |
| RBM25 R | ACACGGTAGGCACTAGGACA |
| Caspase-3 F | GGAGCTTGGAACGCGAAGAA |
| Caspase-3 R | TTGCGAGCTGACATTCCAGT |
| Bax F | TCCGAAGAGGTGGGCTAAGAAG |
| Bax R | CTGCTGCCACAAACATGCAC |
| Bcl-2 F | CATCTCATGCCAAGGGGGAA |
| Bcl-2 R | TATCCCACTCGTAGCCCCTC |
| CSF1 F | TGGACGATCCCGTTTGCTAC |
| CSF1 R | GACACAGGCCTCGTTCTGTT |
| ERK F | ATGCTGGACTGTTGGCAGAA |
| ERK R | GGCCGTGAAGTCTGGGATAG |
| C-FOS F | TTTCAACGCGGACTACGAGG |
| C-FOS R | GCGCAAAAGTCCTGTGTGTT |
| R-EGR1 F | ATCAAAGCCTTCGCCACTCA |
| R-EGR1 R | GTGTAAGCTCATCCGAGCGA |
| PARP1 F | ACCACGCACAATGCCTATGA |
| PARP1 R | AGCAGTCTCCGGTTGTGAAG |
| MAP4K4 F | GACCTCTCAAACCTGCTGTAAGG |
| MAP4K4 R | CCGAGGTGGAATCATCAGCC |
| MAP4K4-a/b F | TCACTACGACCCTGCTGAC |
| MAP4K4-a/b R | TGCTTGGCTATTTTGCTG |
| CSF1 F | CCACTAGCGAGCAAGGAAG |
| CSF1 R | GTCTCCATTTGGCTGTCGAT |
