# Supplementary material for: RBM25 Regulates p38 MAPK Pathway Activation via Exon 16 Skipping of MAP4K4 in a Rat Model of Post‐Infarction Heart Failure
Source: FASEB Bioadv. 2025 Dec 16;7(12):e70074. doi: 10.1096/fba.2025-00201 (PMC12707302; doi:10.1096/fba.2025-00201)
Supplement: Supplementary file 5 — Table S2: Statistical analysis of molecular docking scores (docking score, kcal/mol). [file FBA2-7-e70074-s002.docx]

Table S2: Statistical Analysis of Molecular Docking Scores (Docking Score, kcal/mol)

| Rank | MAP4K4-MAP3K1 | MAP4K4-16-MAP3K1 |
| --- | --- | --- |
| 1 | -589.42 | -590.18 |
| 2 | -509.80 | -557.50 |
| 3 | -506.98 | -546.93 |
| 4 | -496.67 | -514.08 |
| 5 | -495.48 | -506.12 |
