# Supplementary material for: RBM25 Regulates p38 MAPK Pathway Activation via Exon 16 Skipping of MAP4K4 in a Rat Model of Post‐Infarction Heart Failure
Source: FASEB Bioadv. 2025 Dec 16;7(12):e70074. doi: 10.1096/fba.2025-00201 (PMC12707302; doi:10.1096/fba.2025-00201)
Supplement: Supplementary file 6 — Table S3: Changes in rat echocardiographic parameters. [file FBA2-7-e70074-s005.docx]

Table S3: Changes in Rat Echocardiographic Parameters

| Group | Time Point | LVEDD (mm) | LVESD (mm) | LVEF (%) | LVFS (%) |
| --- | --- | --- | --- | --- | --- |
| Sham | Baseline | 4.7 ± 0.3 | 2.9 ± 0.2 | 68.1 ± 2.1 | 38.3 ± 1.5 |
|  | 4 weeks | 4.7 ± 0.3 | 2.9 ± 0.2 | 67.5 ± 2.0 | 38.3 ± 1.4 |
|  | 8 weeks | 4.8 ± 0.4 | 3.0 ± 0.3 | 66.8 ± 2.2 | 37.5 ± 1.6 |
| HF | Baseline | 4.7 ± 0.3 | 2.9 ± 0.2 | 68.5 ± 2.0 | 38.3 ± 1.4 |
|  | 4 weeks | 5.3 ± 0.4*† | 3.4 ± 0.3*† | 64.1 ± 2.5*† | 35.8 ± 1.8*† |
|  | 8 weeks | 5.5 ± 0.4*† | 3.6 ± 0.4*† | 63.0 ± 3.0*† | 34.5 ± 2.0*† |
| OE-NC | Baseline | 4.8 ± 0.4 | 3.0 ± 0.2 | 68.0 ± 2.3 | 37.5 ± 1.6 |
|  | 4 weeks | 5.6 ± 0.4*† | 3.7 ± 0.3*† | 61.4 ± 2.6*† | 33.9 ± 1.9*† |
|  | 8 weeks | 5.8 ± 0.4*† | 3.9 ± 0.4*† | 59.6 ± 3.1*† | 32.8 ± 2.1*† |
| OE-RBM25 | Baseline | 4.6 ± 0.3 | 2.8 ± 0.2 | 69.0 ± 2.1 | 39.1 ± 1.5 |
|  | 4 weeks | 6.0 ± 0.5*† | 4.3 ± 0.4*#† | 50.4 ± 3.0*#† | 28.3 ± 2.2*#† |
|  | 8 weeks | 7.0 ± 0.5*#† | 5.3 ± 0.4*#† | 46.5 ± 3.5*#† | 24.3 ± 2.5*#† |
| OE-RBM25 + SB203580 | Baseline | 4.6 ± 0.3 | 2.8 ± 0.2 | 68.3 ± 2.2 | 39.1 ± 1.6 |
|  | 4 weeks | 6.0 ± 0.4*† | 3.8 ± 0.4*#† | 65.3 ± 2.8*#† | 36.7 ± 2.0*#† |
|  | 8 weeks | 6.7 ± 0.4*† | 4.7 ± 0.4*#† | 53.8 ± 3.2*#† | 29.9 ± 2.2*#† |
| sh-NC | Baseline | 4.5 ± 0.3 | 2.7 ± 0.2 | 69.2 ± 2.4 | 40.0 ± 1.7 |
|  | 4 weeks | 5.4 ± 0.4*† | 3.5 ± 0.3*† | 61.5 ± 2.4*† | 35.2 ± 1.7*† |
|  | 8 weeks | 5.8 ± 0.4*† | 3.9 ± 0.4*† | 59.8 ± 2.9*† | 32.8 ± 1.9*† |
| sh-RBM25 | Baseline | 4.8 ± 0.3 | 2.9 ± 0.2 | 68.5 ± 2.0 | 39.6 ± 1.4 |
|  | 4 weeks | 5.1 ± 0.3#† | 3.3 ± 0.3*† | 64.4 ± 2.4*#† | 35.3 ± 1.7*#† |
|  | 8 weeks | 5.3 ± 0.3*#† | 3.4 ± 0.3*#† | 63.7 ± 2.8*#† | 35.8 ± 1.8*# |
| sh-RBM25 + Gambogic Amide | Baseline | 4.7 ± 0.3 | 2.9 ± 0.2 | 68.7 ± 2.2 | 38.3 ± 1.6 |
|  | 4 weeks | 5.3 ± 0.4*† | 3.6 ± 0.4*† | 59.8 ± 2.8*#† | 32.1 ± 2.0*#† |
|  | 8 weeks | 5.6 ± 0.5*† | 3.9 ± 0.4*#† | 58.6 ± 3.6*#† | 30.4 ± 2.6*#† |

#: P < 0.05 vs. corresponding control group (OE-NC vs. HF; OE-RBM25 vs. OE-NC; OE-RBM25 + SB203580 vs. OE-RBM25; sh-NC vs. HF; sh-RBM25 vs. sh-NC; sh-RBM25 + Gambogic Amide vs. sh-RBM25).

*: P < 0.05 vs. baseline in the same group (comparisons at 4 and 8 weeks vs. baseline).

†: P < 0.05 vs. sham group (at the same time point).

For all groups, n=6; comparisons were performed using one-way ANOVA with Tukey's post-hoc test (intergroup) and paired t-test (intragroup).
