# Supplementary material for: RBM25 Regulates p38 MAPK Pathway Activation via Exon 16 Skipping of MAP4K4 in a Rat Model of Post‐Infarction Heart Failure
Source: FASEB Bioadv. 2025 Dec 16;7(12):e70074. doi: 10.1096/fba.2025-00201 (PMC12707302; doi:10.1096/fba.2025-00201)
Supplement: Supplementary file 7 — Data S1: fba270074‐sup‐0007‐DataS1. [file FBA2-7-e70074-s006.docx]

Supplementary Methods 1：Detailed HF model establishment protocol

Rats were fasted for 6 hours with free access to water before surgery. Anesthesia was induced with 5% isoflurane in 100% oxygen via a nose cone. After loss of pedal withdrawal reflex, endotracheal intubation was performed, and mechanical ventilation was initiated using a rodent ventilator (respiratory rate: 75 breaths/min, tidal volume: 6-8 mL/kg adjusted to body weight, I:E ratio 1:1, with 2 cm H₂O PEEP to prevent alveolar collapse). Anesthesia was maintained with 2% isoflurane delivered through a precision vaporizer. End-tidal CO₂ was monitored and maintained between 35–45 mmHg. A left thoracotomy was performed at the third and fourth intercostal spaces adjacent to the sternum. The ribs were gently retracted using a micro-rib spreader to expose the pericardium. The pericardium was incised to visualize the heart, and the LAD coronary artery was ligated 1-2 mm below the left atrial appendage with a 6-0 polypropylene suture. After the procedure, the heart was returned to the thoracic cavity, and blood and air were evacuated from the pericardial cavity. The thoracic incision was promptly closed, and erythromycin ointment was applied locally for three consecutive days to prevent infection, with postoperative analgesia provided via buprenorphine (0.05 mg/kg) subcutaneously every 12 hours for 72 hours.

Animals meeting any humane endpoint criterion were euthanized within 24 hours via terminal exsanguination under deep anesthesia (5% isoflurane): weight loss >20% over three consecutive days; severe dyspnea (respiratory rate >100 breaths/min or cyanosis); complete immobility (inability to access food/water); sustained hypothermia (rectal temperature <35°C); Signs of severe distress (hunching, tremors,

or vocalization).

Animals meeting any humane endpoint criterion were euthanized within 24 hours via terminal exsanguination under deep anesthesia (5% isoflurane): weight loss >20% over three consecutive days; severe dyspnea (respiratory rate >100 breaths/min or cyanosis); complete immobility (inability to access food/water); sustained hypothermia (rectal temperature <35°C); Signs of severe distress (hunching, tremors, or vocalization).

Supplementary Methods 2: Detailed grouping and intervention protocols

To construct a heart failure model, in vitro RBM25 suppression and overexpression lentiviral vectors were generated and transfected into HEK293T (human embryonic kidney) cells to establish stable expression for subsequent use. After LAD ligation, a total of 12 rats postoperatively died attributed to surgical infections (confirmed by necropsy, n=5) and 7 died due to exacerbated heart failure.

Injections were administered once every four weeks, for a total of two injections. The OE-RBM25 + SB203580 group received intraperitoneal injections of 500 µL of the p38 MAPK inhibitor SB203580 daily for eight consecutive weeks. Similarly, the sh-RBM25 + Gambogic Amide group received intraperitoneal injections of 500 µL of the Gambogic Amide daily for eight weeks. All other groups were administered an equivalent volume of sterile saline intraperitoneally during the same period, with drug-treated rats housed in individually ventilated cages to prevent cross-contamination.

During the intervention, respiratory rate, mobility, and surgical wound integrity was inspected every day. Body weight was monitored weekly to assess overall health and response to treatment.

Supplementary Methods 3: Detailed TTC staining protocol

Following euthanasia, the hearts were promptly excised and immersed in phosphate-buffered saline (PBS) at 0-4℃ for transfer for no more than 30 minutes to prevent ischemic preconditioning artifacts. The hearts were then frozen at -20℃ for 30 minutes to facilitate sectioning, with apical-to-base orientation standardized using a custom slicing matrix. Transverse slices, approximately 2 mm in thickness, were prepared and incubated in 2% 2,3,5-triphenyl tetrazolium chloride (TTC) solution pre-warmed to 37℃ and pH-adjusted to 7.4 at 37℃ in a light-protected water bath for 30 minutes, with negative controls (0.1% sodium azide pre-treatment for 10 minutes) included to confirm assay specificity. The container was gently agitated every 5 minutes during incubation to ensure even staining. After staining, the slices were rinsed with PBS for 3-5 minutes, photographed immediately, and fixed in 10% neutral formalin for 6 hours. The caudal face of each slice was analyzed using a pathological image analysis system by two blinded operators to measure the infarct area and total area. The infarct volume of each slice was calculated as the product of the infarct area and slice thickness, and the total infarct volume was obtained by summing the infarct volumes of all slices.

Supplementary Methods 4: Detailed ELISA protocol(2.5 ELISA quantitative analysis of NT-pro BNP, CRP, IL-6, and TNF-α)

The concentrations of NT-pro BNP, CRP, IL-6, and TNF-α were determined using a validated enzyme-linked immunosorbent assay (ELISA) protocol (ELISA Kit, MEIMIAN, with pre-experiment validation of cross-reactivity rates (<1% between analytes) and lower limits of detection (LLOD: NT-pro BNP 5 pg/mL, CRP 0.2 ng/mL, IL-6 1.5 pg/mL, TNF-α 2 pg/mL). Serum samples were centrifuged at 2000 × g for 15 minutes at 4℃ within 30 minutes of collection and stored at -80℃ in aliquots to avoid freeze-thaw cycles. Serially diluted standards of NT-pro BNP, CRP, IL-6, and TNF-α were prepared in pooled sham group serum to match matrix effects and loaded into designated wells of an enzyme-coated microplate at 50 μL/well. For test samples, 25 μL of sample diluent with 2-fold dilution was first added to each well, followed by 25 μL of internal quality control (IQC) samples (Bio-Rad Liquichek) every 10 test wells to monitor inter-assay variability, followed by 25 μL of the test sample, ensuring precise dispensing to the bottom of the well without contacting the walls. Plates were read in triplicate, with coefficient of variation (CV) < 8% as acceptance criteria. Plates were agitated for uniform mixing, sealed with adhesive film, and incubated at 37℃ for 30 minutes under controlled conditions. After incubation, the sealing film was removed, and the liquid was discarded. Wells were washed using a washing buffer prepared by diluting the concentrated solution (1:20) with distilled water. Each well was filled completely with washing buffer, allowed to stand for 30 seconds, and then emptied. This washing process was repeated five times, with the plate tapped dry after the final wash. Subsequently, 50 μL of enzyme-labeled conjugate was added to each well, excluding blank control wells. The plate was resealed and incubated at 37℃ for 30 minutes. Following this incubation, the sealing film was removed, and the washing procedure was repeated five times as previously described. Subsequently, 50 μL of substrate solution A and B were sequentially added to each well. The plate was agitated for thorough mixing and incubated at 37℃ in the dark for 10 minutes to allow color development. The reaction was terminated by adding 50 μL of stop solution to each well. Optical density (OD) was measured at 450 nm using a microplate reader within 15 minutes of reaction termination.

Supplementary Methods 5: Detailed WB protocol(2.7 Western blotting (WB) analysis)

Myocardial tissue samples were homogenized in ice-cold lysis buffer and incubated on ice for 30 min with vortexing every 2 min. Lysates were centrifuged at 14,000 × g for 15 min at 4°C. Supernatants were filtered through 0.22-μm PVDF syringe filters to remove debris. Protein concentration was determined using the BCA assay kit (Beyotime, P0012) with BSA standards (0.5–10 μg/μL, R² ≥ 0.98). Samples were diluted to fall within the linear range. A total of 80 μg protein per sample was mixed with 20 μL 5× loading buffer (supplemented with 5% β-mercaptoethanol), denatured at 95°C for 5 min, and resolved by SDS-PAGE. Proteins were transferred to PVDF membranes, hydrated in 1× TBST, and blocked in 5% skim milk for 40 min at RT. The membranes were incubated overnight at 4℃ with the following primary antibodies: RBM25 (Proteintech, 25297-1-AP, 1:2000), Caspase-3 (Abcam, ab184787, 1:2000), Bax (Abcam, ab32503, 1:1000), Bcl-2 (Abcam, ab182858, 1:1000), CSF1 (Huabio, DF12536, 1:5000), ERK (Affinity, AF0155, 1:1000), p-ERK (Affinity, BF8004, 1:1000), c-FOS (Affinity, AF5354, 1:1000), EGR1 (Affinity, AF0589, 1:1000), PARP1 (Affinity, DF7198, 1:1000), GAPDH (Servicebio, GB11002, 1:5000). After TBST washes, membranes were incubated with HRP-conjugated secondary antibodies: Goat anti-rabbit IgG (Servicebio, GB23303, 1:3000), Goat anti-mouse IgG (Servicebio, GB23301, 1:5000) for 40 minutes at room temperature. Protein signals were visualized using a Bio-Rad ChemiDoc XRS+ chemiluminescence imaging system, and the resulting images were saved for analysis. Densitometric analysis of protein bands was performed using ImageJ software (version 1.8.0). Relative protein expression levels were calculated by normalizing the grayscale intensity of the target protein band to that of the internal control.

Supplementary Methods 6: Detailed TUNEL protocol(2.8 TUNEL assay for detection of cardiomyocyte apoptosis)

Myocardial tissue samples were paraffin-embedded, sectioned, and subjected to standard deparaffinization and rehydration procedures. Following these steps, the sections were washed twice with PBS. Proteinase K working solution was prepared by diluting the stock solution (200 µg/mL) with PBS at a 1:9 ratio to achieve a final concentration of 20 µg/mL. A total of 100 µL of Proteinase K working solution was applied to each section, ensuring complete coverage of the tissue. The sections were incubated at 37℃ for 20 minutes, followed by three washes with PBS. During the incubation, the TUNEL reaction mixture was prepared by combining reaction solution 1 and 2 at a 1:9 ratio. For each tissue section, 50 µL of the TUNEL reaction mixture was added to cover the tissue, and the sections were incubated at 37℃ for 1 hour. After incubation, the sections were washed three times with PBS and stained with DAPI for 10 minutes at room temperature. To prevent drying, a drop of PBS was added to each slide before imaging. Fluorescence microscopy was used to observe the sections, with images captured from 3–5 randomly selected fields per section under 400 magnifications. (TUNEL Assay Kit: TMR (red) Tunel Cell Apoptosis Detection Kit, Servicebio, G1502-50T).

Supplementary Methods 7: Detailed docking protocol(2.11 HDOCK Molecular Docking Simulations)

Structural preprocessing was performed sequentially: Protonation State Assignment: Structures were processed through H++ 3.0 web server at pH 7.0 to assign physiological protonation states. Charge Parameterization: Using UCSF Chimera 1.17 , Amber ff14SB force field charges were assigned. Protein-protein docking was executed on the HDOCK server under these conditions: Docking Mode: Fast Fourier transform (FFT)-based rigid-body docking Scoring Function: ITScorePP Output: Top 100 poses ranked by docking score (negative values indicate favorable binding, with lower scores indicating higher affinity). The top five poses were subjected to binding interface analysis. Structural visualization was performed using PyMOL 2.5.7 (Schrödinger, LLC., 2023), and interaction diagrams were generated with LigPlot+ v.2.2.5 using default parameters (hydrogen bond cutoff: 3.5 Å, hydrophobic contact cutoff: 4.0 Å).
